# Human infections with avian influenza A(H5) viruses with potential pandemic risk: 1997–2025

**DOI:** 10.1093/nsr/nwaf471

**Published:** 2025-11-10

**Authors:** Wei Wang, Jiabao Xing, Hui Jiang, Fayu Lu, Haoxuan Huang, Yuqian Zhang, Andi Sun, Yi Han, Jian Lu, Benjamin J Cowling, Peter Horby, Richard J Webby, Timothy M Uyeki, Hongjie Yu

**Affiliations:** School of Public Health, Fudan University, Key Laboratory of Public Health Safety, Ministry of Education, Shanghai 200032, China; School of Public Health, Fudan University, Key Laboratory of Public Health Safety, Ministry of Education, Shanghai 200032, China; Beijing Chest Hospital, Capital Medical University, Beijing 100071, China; School of Public Health, Fudan University, Key Laboratory of Public Health Safety, Ministry of Education, Shanghai 200032, China; School of Public Health, Fudan University, Key Laboratory of Public Health Safety, Ministry of Education, Shanghai 200032, China; School of Public Health, Fudan University, Key Laboratory of Public Health Safety, Ministry of Education, Shanghai 200032, China; School of Public Health, Fudan University, Key Laboratory of Public Health Safety, Ministry of Education, Shanghai 200032, China; School of Public Health, Fudan University, Key Laboratory of Public Health Safety, Ministry of Education, Shanghai 200032, China; State Key Laboratory of Protein and Plant Gene Research, Center for Bioinformatics, School of Life Sciences, Peking University, Beijing 100871, China; School of Public Health, Li Ka Shing Faculty of Medicine, The University of Hong Kong, Hong Kong 999077, China; Pandemic Sciences Institute, Nuffield Department of Clinical Medicine, University of Oxford, Oxford OX3 7DQ, UK; St Jude Children’s Research Hospital, Memphis, TN 38105, USA; Influenza Division, National Center for Immunization and Respiratory Diseases, Centers for Disease Control and Prevention, Atlanta, GA 30329, USA; School of Public Health, Fudan University, Key Laboratory of Public Health Safety, Ministry of Education, Shanghai 200032, China; Shanghai Institute of Infectious Disease and Biosecurity, Fudan University, Shanghai 200032, China; Department of Infectious Diseases, Huashan Hospital, Fudan University, Shanghai 200032, China

**Keywords:** Avian influenza A(H5) viruses, zoonotic infection, phylodynamics, epidemiology, disease severity, transmission dynamics

## Abstract

Highly pathogenic avian influenza (HPAI) A(H5) viruses have caused sporadic human infections since 1997, with recent detections in the Americas and Asia. However, the evolutionary dynamics of different HPAI A(H5) viruses at the animal–human interface, along with their associated disease severity, propensity for animal-to-human (zoonotic) spillover, and human-to-human transmission potential, remain unclear. Here, we combine available genetic and epidemiological data with mechanistic models to better understand the global spread of HPAI A(H5) viruses that spilled over to humans in 1997–2025. Analysis of 7445 subsampled hemagglutinin gene sequences revealed frequent regional succession of HPAI A(H5) virus clades that varied by geographic location. The 1104 reported human HPAI A(H5) cases exhibited subtype- and clade-specific heterogeneity in age, gender, and exposure sources (*p* < 0.001). After adjusting for under-reporting, we estimated case-fatality risk to be low for HPAI A(H5N1) clade 2.3.4.4b (0.7%, 95%CI: 0.02%–3.9%) and for A(H5N6) clades 2.3.4x (0%, 0%–1.1%) and 2.3.4.4b (1.6%, 0.7%–3.2%), compared with other A(H5) clades (range: 4.7%–15.0%). We also show that, while the transmissibility of HPAI A(H5) viruses between humans remains very low to date (mean *R*_t_: 0.10–0.23), zoonotic transmission has increased with the emergence of bovine-origin clade 2.3.4.4b (incidence: 7.85 per million people per year), relative to other avian-origin A(H5) clades (range: 1.54–5.04 per million people per year). Although other factors such as exposure sources, routes of transmission, immune function, underlying medical conditions, and clinical management can influence outcomes of case-patients, these findings highlight the ongoing pandemic threat posed by HPAI A(H5) viruses and the need for ongoing comprehensive surveillance, genotypic and phenotypic characterization, and preparedness.

## INTRODUCTION

Highly pathogenic avian influenza (HPAI) A(H5) viruses that are currently circulating worldwide among wild birds and poultry are derived from the A/goose/Guangdong/1/96 (Gs/Gd) lineage that was first isolated from a goose in Guangdong Province, China, in 1996 [1]. The first known human case of HPAI A(H5N1) virus infection was identified in Hong Kong SAR, China, in May 1997 [2]; 17 additional human HPAI A(H5N1) cases were virologically confirmed during 1997 before the outbreak was brought under control [3]. Although controlled in Hong Kong SAR, China, viruses of the Gs/Gd lineage continued to spread among poultry in the region until a dramatic increase in HPAI A(H5N1) virus activity and distribution was observed starting in late 2003 (number of countries being affected: 8 [4]), with subsequent spread through migratory birds and poultry movement across Asia, Europe and Africa [3,5]. From 2008 onwards, several novel HPAI A(H5) subtype viruses were detected in wild birds and poultry, including HPAI A(H5N2), A(H5N3), A(H5N5), A(H5N6) and A(H5N8) viruses, with A(H5N6) and A(H5N8) viruses reported in poultry and humans in China and Russia, respectively [6].

Notably, the predominant clade 2.3.4.4b HPAI A(H5) viruses, detected widely in wild birds, poultry, and various mammalian species across much of the world, were spread by wild birds from Europe to North America in late 2021 [1,7]. Clade 2.3.4.4b A(H5N1) viruses of the B3.13 and D1.1 genotypes have been detected in dairy cattle in addition to poultry in the United States, infecting 1081 dairy herds cumulatively in 18 states through 27 October 2025 [8].

Since the first detected instance of presumed cow-to-human transmission in the United States in late March 2024 [9,10], a sharp increase occurred in human cases of HPAI A(H5N1) virus infection [11]. While spatiotemporal variation in case reporting rate may be substantial, the officially reported number of new cases during 2024–2025 was nearly double the cumulative number of cases recorded during the previous 9 years (*N* = 36) [11,12]. Most HPAI A(H5N1) cases identified in the United States during 2024–2025 have been associated with exposure to dairy cattle (41/70, 58.6%), with conjunctivitis as the predominant clinical finding [9,12,13]. In other countries, most HPAI A(H5) cases detected during 2003–2024 were associated with poultry exposures, including A(H5N1), A(H5N6) and A(H5N8) virus infections [14,15]. Unlike human HPAI A(H5) cases reported before the emergence of bovine-origin HPAI A(H5N1) clade 2.3.4.4b viruses, the clinical severity of HPAI A(H5N1) cases in the United States, regardless of exposure source, has been predominantly mild (hospitalization proportion: 4/70) [16]. While abundant genetic sequencing data exist on HPAI A(H5) viruses [17,18], their evolutionary trajectories at the animal–human interface remain poorly defined, with previous studies focusing primarily on the domestic bird–wild bird interface [5,19]. Furthermore, accurate characterization of the long-term epidemiological profile, disease severity and transmission potential of human infections with different HPAI A(H5) virus subtypes and clades is hindered by a lack of a standardized global database of individual case data and by under-reporting driven by variable surveillance quality.

We systematically collected and analyzed available human HPAI A(H5) case data reported to the World Health Organization (WHO) and national health authorities (Fig. S1), along with the available virus sequence data from 1 May 1997, through 31 July 2025. We assessed the evolutionary dynamics of different subtypes and clades of HPAI A(H5) viruses at the animal–human interface since 1997. Leveraging detailed case incidence data, we used mathematical models to estimate disease severity, zoonotic spillover and human-to-human transmissibility of HPAI A(H5) viruses, while accounting for heterogeneity in surveillance quality.

## RESULTS

### Genomic epidemiology of zoonotic HPAI A(H5) cases

**Figure 1.**
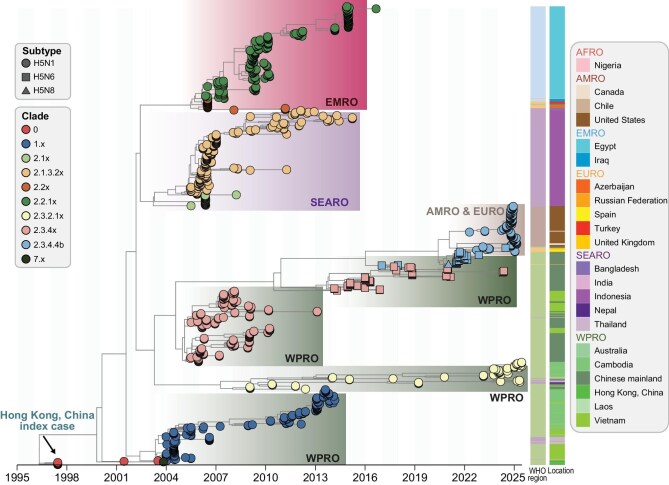
Time-scaled maximum-likelihood tree of HPAI A(H5) virus HA sequences from human cases worldwide. The phylogeny is based on all available sequences of viruses from human cases (*N* = 838). Each point, square and triangle indicates an HA subtype, while branch tips denote pre-defined major virus clades. Phylogenetic clusters of human HPAI A(H5) cases are defined by their major spatiotemporal distribution and annotated with shaded areas and text. The location and WHO region of each isolation are shown as colored bars on the right. The box colors indicate the WHO regions of the isolates. Abbreviations for WHO regions: AFRO, Africa Region; AMRO, Americas Region; EMRO, Eastern Mediterranean Region; EURO, Europe Region; SEARO, South-East Asia Region; WPRO, Western Pacific Region. HA = hemagglutinin gene; HPAI = highly pathogenic avian influenza.

**Figure 2.**
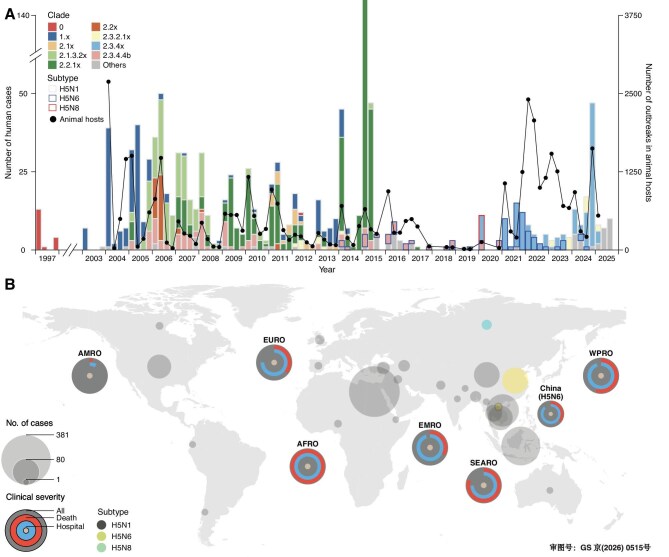
Spatiotemporal distribution of human HPAI A(H5) cases worldwide. (A) Time series of human HPAI A(H5N1), A(H5N6) and A(H5N8) cases by epidemic months. Each bar represents the total number of reported cases by virus subtype and clade at 3-month intervals, while each black point indicates the number of A(H5) animal outbreaks over the same time period. (B) Regional differences in the cumulative number of human HPAI A(H5N1), A(H5N6), and A(H5N8) cases and associated disease severity. The size of grey bubbles indicates the cumulative number of reported cases in such a location. Observed regional disease severity is presented as a circular bar plot, where blue and red bars represent the regional proportions of hospitalized and fatal cases, respectively. Since seven human A(H5N8) cases were asymptomatic, their disease severity distributions were not included. HPAI = highly pathogenic avian influenza.

Overall, the detection frequency of HPAI A(H5N1), A(H5N6) and A(H5N8) virus hemagglutinin (HA) gene sequences increased over time [*N* = 40 988; hereafter referred to as A(H5)], with a significant rise observed during 2014–2018 (*N* = 7603) and again since 2021 (*N* = 27 123) (Fig. S2A). The inferred phylogenetic tree, based on 7445 subsampled HPAI A(H5) virus sequences from multiple hosts (subsampling for computational efficiency, see Supplementary Text for details), revealed frequent viral clade turnovers followed by cross-regional transmission, which is consistent with a previous report [5]. These patterns were subsequently reflected in sequences obtained from clinical specimens of human cases (human case-derived sequences, *N* = 838) (Fig. 1, Fig. S2B). Human case-derived HPAI A(H5) virus clades were detected only after the same clade had first been detected in animal hosts, with an observed mean time difference of 66 (IQR: 24–88) months between reported detections of animal- and human-derived sequences of the same clade (Fig. S3). This finding was further supported by a temporal correlation between the numbers of reported human HPAI A(H5) cases and outbreaks in animal hosts (*r_s_* = 0.26, *P*-value = 0.024) (Fig. 2A). Additionally, we found geographical dominance of HPAI A(H5) viruses in both animal hosts and humans, with the latter showing greater predominance in distinct geographical regions (Fig. 1, Fig. S2B).

We then focused on the geographic spread of HPAI A(H5) viruses responsible for sporadic human infections. Clade 0 viruses that caused human infections in Hong Kong SAR, China, in 1997 clustered independently before being succeeded by clade 1 viruses that caused human infections during 2003–2014 (Fig. 1, Figs S2B and S4). Specifically, clade 1 viruses were first detected in humans in Hong Kong SAR, China, who had traveled to southern China in 2003, and was the dominant lineage in Vietnam, Thailand and Cambodia during 2004–2006 (Fig. 1, Figs S2B and S4). Clade 2 viruses, which further diversified into five major phylogenetically distinct A(H5N1) HA clusters with human infections (2.1, 2.2, 2.3, 2.3.2.1x and 2.3.4.4b), maintained comparable clade-specific geographical signatures across human cases. Among these, clades 2.1x and 2.1.3.2x predominantly caused human infections in Indonesia during 2005–2007, with the latter continuing to predominate until 2016. Clade 2.2x viruses caused human infections in Azerbaijan, Iraq and Turkey in 2005–2006, before clade 2.2.1 viruses emerged and became the predominant lineage in Egypt from 2006 to 2015. Within descendants of clade 2.3 viruses, the earliest emerging clade 2.3.4x was predominant only in China and Vietnam during 2005–2013. Between 2013 and 2016, A(H5N6) and A(H5N8) viruses from this clade continued to circulate among wild birds and poultry exclusively in the Western Pacific and Europe. In contrast, clade 2.3.2.1x has been the predominant lineage that has caused human infections in eight countries across three WHO regions (including the Western Pacific, South-East Asia and the Americas) from 2008 to 2025 (Fig. 1, Figs S2B and S4). Currently, in addition to clade 2.3.2.1x, clade 2.3.4.4b viruses, containing different NA subtypes including N1, N6 and N8, have emerged as the dominant lineage in Europe, the Western Pacific and the Americas.

### Epidemiology of human HPAI A(H5) cases

As of 31 July 2025, a cumulative total of 1104 HPAI A(H5) cases were reported worldwide, including 1009 A(H5N1) cases, 88 A(H5N6) cases and 7 A(H5N8) cases. Information on 226 (20.5%) of 1104 HPAI A(H5) cases was provided by national health agencies, while data on the remaining 878 (79.5%) cases were collected from WHO and other publicly available sources (Table S2). No statistically significant differences were found in the completeness of demographic, key timeline, or epidemiological exposure variables between data provided by the national health agencies and data obtained from publicly available sources (*P*-value = 0.282), indicating good representativeness of multi-source case data used in the current study. Despite variable numbers of reported human HPAI A(H5) cases caused by different virus subtypes and clades worldwide, 87.8% of illness onset (*N* = 969) occurred in countries in the Northern Hemisphere, where epidemic peaks were more frequently observed in January–March and September–December, corresponding to the winter–spring seasons and spring festival periods in these regions (Fig. 2A, Fig. S5). Unlike the widespread distribution of reported HPAI A(H5N1) cases, nearly all HPAI A(H5N6) and A(H5N8) cases were reported from China (*N* = 87, 98.9%) and Russia (*N* = 7, 100%), respectively (Fig. 2B).

Demographic data on both age and gender were available for 1049 (95.0%) of the reported 1104 HPAI A(H5) cases studied (Table 1). The age distribution was younger for HPAI A(H5N1) cases (median: 18 years, IQR: 5–32 years), compared with HPAI A(H5N6) cases (median: 50 years, IQR: 35–57 years, *P*-value < 0.001) and HPAI A(H5N8) cases (range: 29–60 [20]). No sex-based differences were observed across the three HPAI A(H5) virus subtypes, with male-to-female ratios of 0.93 for A(H5N1), 1.26 for A(H5N6) and 0.40 for A(H5N8) cases (*P*-value = 0.237) (Table 1). Following the emergence of the HPAI A(H5N1) virus, clade 2.3.4.4b, the primary sources of virus exposure for human HPAI A(H5N1) cases were from dairy cattle and commercial poultry in the United States (before and during culling operations) (92.8%, 65/70). This contrasts with cases associated with HPAI A(H5N6) and A(H5N8) viruses (95.6%–100%) and other HPAI A(H5N1) virus clades (90.8%–100%), where the primary sources of virus exposure were to backyard poultry and their contaminated environments or at live poultry markets (*P*-value < 0.001).

**Table 1.** Epidemiological characteristics of reported cases of highly pathogenic avian influenza A(H5) virus infection worldwide, May 1997–July 2025.

		H5N1 (*N* = 1009)						H5N6 (*N* = 88)		H5N8 (*N* = 7)
Characteristics	Total (*N* = 1104)	2.2x & 2.2.1x (*N* = 371)	2.1x & 2.1.3.2x (*N* = 200)	1.x (*N* = 181)	2.3.4.4b (*N* = 84)	2.3.4x (*N* = 73)	Others^a^ (*N* = 100)	2.3.4.4b (*N* = 64)	2.3.4x (*N* = 24)	2.3.4.4b (*N* = 7)
Sex										
Male	513 (46.5)	151 (40.7)	87 (43.5)	92 (50.8)	42 (50.0)	37 (50.7)	53 (53.0)	38 (59.4)	11 (45.8)	2 (28.6)
Female	536 (48.6)	218 (58.8)	92 (46.0)	89 (49.2)	15 (17.9)	36 (49.3)	42 (42.0)	26 (40.6)	13 (54.2)	5 (71.4)
Unknown	55 (5.0)	2 (0.5)	21 (10.5)	0 (0)	27 (32.1)	0 (0)	5 (5.0)	0 (0)	0 (0)	0 (0)
Age										
Median (years, interquartile range)^b^	18 (4–32)	17 (4–32)	20 (9–29)	16 (6–30)	38 (16–53)	25 (16–32)	16 (4–34)	53 (45–58)	39 (26–45)	29–60^c^
0–17	443 (40.1)	185 (49.9)	84 (42.0)	96 (53.0)	6 (7.1)	21 (28.8)	41 (41.0)	6 (9.4)	4 (16.7)	0 (0)
18–64	602 (54.5)	180 (48.5)	93 (46.5)	81 (44.8)	71 (84.5)	50 (68.5)	54 (54.0)	47 (73.4)	19 (79.2)	7 (100)
≥65	29 (2.6)	4 (1.1)	1 (0.5)	4 (2.2)	2 (2.4)	1 (1.4)	5 (5.0)	11 (17.2)	1 (4.2)	0 (0)
Unknown	30 (2.7)	2 (0.5)	22 (11.0)	0 (0)	5 (6.0)	1 (1.4)	0 (0)	0 (0)	0 (0)	0 (0)
Clinical constellations										
With any symptoms	238 (21.6)	0 (0)	0 (0)	0 (0)	74 (88.1)	39 (53.4)	40 (40.0)	63 (98.4)	22 (91.7)	0 (0)
Only non-conjunctival symptoms	185 (77.7)				21 (28.4)	39 (100)	40 (100)	63 (100)	22 (100)	
Conjunctivitis only	26 (10.9)				26 (35.1)	0 (0)	0 (0)	0 (0)	0 (0)	
Conjunctivitis and respiratory symptoms	14 (5.9)				14 (18.9)	0 (0)	0 (0)	0 (0)	0 (0)	
Conjunctivitis and non-respiratory symptoms	13 (5.5)				13 (17.6)	0 (0)	0 (0)	0 (0)	0 (0)	
Without any symptoms	15 (1.4)	0 (0)	0 (0)	0 (0)	6 (7.1)	0 (0)	2 (2.0)	0 (0)	0 (0)	7 (100)
Unknown	851 (77.1)	371 (100)	200 (100)	181 (100)	4 (4.8)	34 (46.6)	58 (58.0)	1 (1.6)	2 (8.3)	0 (0)
Hospitalization										
Yes	923 (83.6)	362 (97.6)	160 (80.0)	168 (92.8)	10 (11.9)	71 (97.3)	76 (76.0)	54 (84.4)	22 (91.7)	0 (0)
No	83 (7.5)	1 (0.3)	0 (0)	1 (0.6)	67 (79.8)	1 (1.4)	3 (3.0)	1 (1.6)	2 (8.3)	7 (100)
Unknown	98 (8.9)	8 (2.2)	40 (20.0)	12 (6.6)	7 (8.3)	1 (1.4)	21 (21.0)	9 (14.1)	0 (0)	0 (0)
Outcome^d^										
Died	511 (46.3)	123 (33.2)	168 (84.0)	101 (55.8)	2 (2.4)	46 (63.0)	40 (40.0)	49 (73.1)	8 (30.8)	0 (0)
Recovered	574 (52.0)	248 (66.8)	32 (16.0)	78 (43.1)	81 (96.4)	27 (37.0)	50 (50.0)	18 (26.9)	18 (69.2)	7 (100)
Unknown	19 (1.7)	0 (0)	0 (0)	2 (1.1)	1 (1.2)	0 (0)	10 (10.0)	0 (0)	0 (0)	0 (0)
**Exposure history**										
Any exposure to animals										
Yes	955 (86.5)	350 (94.3)	146 (73.0)	149 (82.3)	81 (96.4)	64 (87.7)	85 (85.0)	51 (79.7)	22 (91.7)	7 (100)
No	23 (2.1)	1 (0.3)	2 (1.0)	15 (8.3)	0 (0)	0 (0)	2 (2.0)	2 (3.1)	1 (4.2)	0 (0)
Unknown	126 (11.4)	20 (5.4)	52 (26.0)	17 (9.4)	3 (3.6)	9 (12.3)	13 (13.0)	11 (17.2)	1 (4.2)	0 (0)
**Exposure to infected or potentially infected animals**										
Yes										
Poultry	878 (79.5)	350 (94.3)	136 (68.0)	146 (80.7)	39 (46.4)	56 (76.7)	81 (81.0)	42 (65.6)	21 (87.5)	7 (100)
Dairy cows	41 (3.7)	0 (0)	0 (0)	0 (0)	41 (48.8)	0 (0)	0 (0)	0 (0)	0 (0)	0 (0)
No	35 (3.2)	0 (0)	4 (2.0)	15 (8.3)	1 (1.2)	8 (11.0)	5 (5.0)	0 (0)	2 (8.3)	0 (0)
Unknown	150 (13.6)	21 (5.7)	60 (30.0)	20 (11.0)	3 (3.6)	9 (12.3)	14 (14.0)	22 (34.4)	1 (4.2)	0 (0)
Visited live poultry market										
Yes	111 (10.1)	12 (3.2)	19 (9.5)	5 (2.8)	0 (0)	22 (30.1)	26 (26.0)	13 (20.3)	14 (58.3)	0 (0)
No	354 (32.1)	85 (22.9)	40 (20.0)	64 (35.4)	81 (96.4)	25 (34.2)	46 (46.0)	0 (0)	6 (25.0)	7 (100)
Unknown	639 (57.9)	274 (73.9)	141 (70.5)	112 (61.9)	3 (3.6)	26 (35.6)	28 (28.0)	51 (79.7)	4 (16.7)	0 (0)
Exposure to backyard poultry										
Yes	243 (22.0)	75 (20.2)	37 (18.5)	43 (23.8)	5 (6.0)	22 (30.1)	35 (35.0)	18 (28.1)	8 (33.3)	0 (0)
No	225 (20.4)	22 (5.9)	21 (10.5)	22 (12.2)	76 (90.5)	25 (34.2)	40 (40.0)	0 (0)	12 (50.0)	7 (100)
Unknown	636 (57.6)	274 (73.9)	142 (71.0)	116 (64.1)	3 (3.5)	26 (35.6)	25 (25.0)	46 (71.9)	4 (16.7)	0 (0)
Belongs to an A(H5) case cluster										
Yes	177 (16.0)	40 (10.8)	45 (22.5)	33 (18.2)	26 (31.0)	5 (6.8)	19 (19.0)	2 (3.1)	0 (0)	7 (100)
No	927 (84.0)	331 (89.2)	155 (77.5)	148 (81.8)	58 (69.0)	68 (93.2)	81 (81.0)	62 (96.9)	24 (100)	0 (0)

a This includes 19 reported cases with clade 0, 4 cases with clade 2.3.2, 21 cases with clade 2.3.2.1x, 2 cases with clade 7 virus infections, and 54 cases without virus clade information. ^b^This is based on 937 individuals with exact ages. ^c^Given data availability, only the age range of 7 A(H5N8) case patients is shown here. ^d^Clinical outcomes for A(H5N6) case patients were obtained from WHO reports.

### Disease severity of human HPAI A(H5) cases

We next investigated disease severity in reported human cases caused by HPAI A(H5) viruses, with a specific focus on examining whether bovine-origin clade 2.3.4.4b infections of humans (reported case-fatality of 0.9% [16]) exhibit a lower disease severity compared with other clades (53.5% [14]) as previously reported. We found individual age, gender, virus subtypes and clades, and the quality of surveillance system (reflecting the capacity to ascertain underlying human cases) to be significantly associated with fatal outcome in multivariable analysis using a generalized linear mixed model with country-level random effects (Table S3). In particular, cases reported through surveillance systems since the start of the COVID-19 pandemic in 2020 had 1.83- to 9.39-fold lower odds of death (*P*-value < 0.05). Human cases with the N6 neuraminidase subtype had 6.30-fold higher odds of death (95% CI: 2.36–16.84) compared to those with the N1 subtype (*P*-value < 0.001). Moreover, compared to cases associated with the bovine-origin HPAI A(H5N1) virus, clade 2.3.4.4b, those associated with avian-origin HPAI A(H5) virus clades show significantly higher odds of death (range: 4.06–104.89), except for cases linked to avian-origin HPAI A(H5N1) virus, clade 2.3.4.4b (OR = 0.37, 95% CI: 0.12–1.15; *P*-value = 0.087).

**Figure 3.**
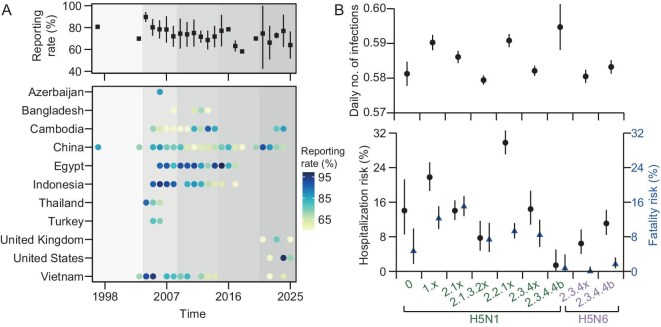
Trends in ascertainment for human HPAI A(H5) cases and their application to disease severity estimates. Each point, square or triangle with error bar shows the point estimate and 95% confidence interval. (A) Estimated year- and country-specific case ascertainment. Grey shaded area denotes different levels of disease surveillance quality in 1997–2025, defined by global responses to major public health emergencies: the SARS outbreaks in 2003, the H1N1 pandemic in 2009, the Ebola outbreaks in 2014, and the COVID-19 pandemic since 2020. (B) Estimated mean daily number of infections (upper panel), case hospitalization and case fatality risks (lower panel) by HPAI A(H5) virus subtype and clade. In the lower panel of (B), black points or blue triangles with error bars show the estimated mean case hospitalization and fatality risks and corresponding 95% confidence intervals. HPAI = highly pathogenic avian influenza.

Given the observed impact of surveillance quality on estimated disease severity, we then used case incidence data to fit our previously proposed Bayesian Poisson model [21] to correct for potential under-reporting of HPAI A(H5) cases when determining clade-specific disease severity (Fig. 3). We estimated that the HPAI A(H5) case reporting varied across countries and years (mean: 73.9%, range: 57.3%–98.1%), with the best surveillance performance observed in Egypt in 2015 (98.1%, 95% CI: 93.7%–99.8%) and in the United States during 2024 (96.8%, 95% CI: 89.7%–99.6%) (Fig. 3A). Moreover, we found that the timing of improvements in case reporting broadly aligned with global responses to infectious disease outbreaks, including the SARS outbreaks in 2003, the 2009 H1N1 pandemic, Ebola outbreaks in 2014, and the COVID-19 pandemic since 2020. We observed that the estimated mean daily number of infections was slightly higher for the bovine-origin HPAI A(H5N1) virus, clade 2.3.4.4b (mean: 0.595, 95% CI: 0.588–0.601) than for other HPAI A(H5N1) and A(H5N6) virus clades (mean: 0.587, 95% CI: 0.512–0.661; *P*-value = 0.019) (Fig. 3B). We further determined HPAI A(H5) virus clade-specific case hospitalization and fatality risk based on the reported number of hospitalizations and deaths, together with the model-based total number of infections. We estimated a relatively lower risk of fatal outcomes for cases with HPAI A(H5N1) virus, clade 2.3.4.4b (case-fatality risk: 0.7%, 95% CI: 0.02%–3.9%), A(H5N6) virus, clade 2.3.4x (0%, 95% CI: 0%–1.1%) and 2.3.4.4b (1.6%, 95% CI: 0.7%–3.2%) than for other virus clades (range: 4.7%–15.0%) (Fig. 3B). These estimates differ from those derived directly from surveillance data, as exemplified by the observed case fatality risks of 3.5% (0.7%–9.9%) for HPAI A(H5N1) clade 2.3.4.4b, and 28.1% (95% CI: 17.6%–40.8%) and 75.9% (95% CI: 56.5%–89.7%) for A(H5N6) clades 2.3.4.4b and 2.3.4x, respectively.

### Transmission potential of HPAI A(H5) viruses in humans

**Figure 4.**
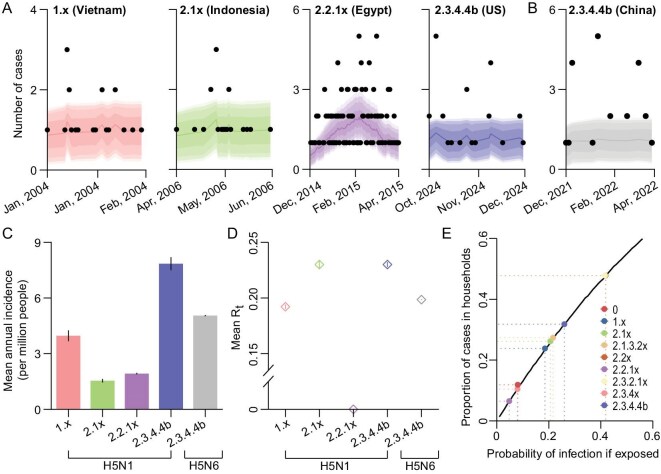
Transmission parameter estimates and model fit for human HPAI A(H5) cases across different virus subtypes and clades. (A) Comparison of reported and estimated mean daily number of cases infected with various HPAI A(H5N1) virus clades and (B) A(H5N6) virus clade in selected study locations. In both panels, the solid line indicates the estimated median of case numbers, while black points represent the reported case numbers. The shaded bands, from dark to light, denote the 80%, 90% and 95% credible intervals for the time-specific case numbers. (C) Model-based annual incidence (per million people) of human HPAI A(H5) virus infections attributable to animal-to-human transmission. Each bar with error bar indicates the mean incidence and 95% confidence interval. (D) Estimated clade-specific *R*_t_ for human-to-human transmission of HPAI A(H5N1) and A(H5N6) viruses. The diamond and inner vertical line show the mean *R*_t_ and corresponding 95% confidence interval. (E) Estimated probability of HPAI A(H5N1) virus infection given exposure to a human case within households of different sizes. In (A) and (C–D), only bovine-origin A(H5N1) virus, clade 2.3.4.4b was included in the analyses. HPAI = highly pathogenic avian influenza.

To understand animal-to-human and human-to-human transmission intensity of HPAI A(H5) virus clades that caused large outbreaks of human cases, we used their case incidence data to fit a time-discrete SIR model with time-varying transmission rates (Fig. 4). We found that this model was able to reconstruct the observed number of cases well in the five selected study locations. These include HPAI A(H5N1) outbreaks in Vietnam in 2004 (observed vs estimated number of cases: 21 vs 20, 95% CrI: 16–32), Indonesia in 2006 (20 vs 19, 95% CrI: 16–32), Egypt in 2014–2015 (160 vs 161, 95% CrI: 102–235), the United States in 2024 (31 vs 32, 95% CrI: 16–32), as well as an HPAI A(H5N6) outbreak in China in 2022 (23 vs 18, 95% CrI: 10–20) (Fig. 4A and B). With this validated model, we further estimated the mean annual incidence of cases attributable to animal-to-human transmission, with the highest incidence observed for bovine-origin HPAI A(H5N1) virus, clade 2.3.4.4b (7.85, 95% CI: 7.48–8.21 per million people per year). This was followed by A(H5N6) virus, clade 2.3.4.4b (5.04, 95% CI: 5.00–5.07 per million people per year), A(H5N1) virus clades 1.x (3.98, 95% CI: 3.69–4.27 per million people per year), 2.2.1x (1.93, 95% CI: 1.88–1.97 per million people per year) and 2.1x (1.54, 95% CI: 1.45–1.64 per million people per year) (Fig. 4C).

Regarding human-to-human transmissibility, no substantial increase in the mean *R*_t_ was observed for HPAI A(H5) viruses before or after the emergence of bovine-origin HPAI A(H5N1) virus, clade 2.3.4.4b (mean: 0.19, range: 0.10–0.23) (Fig. 4D), regardless of virus subtype or clade. Based on 124 possible secondary cases, including 9 (7.3%) with confirmed human contacts only and 115 (92.7%) with exposures to both human cases and infected animal populations, we found no substantial increase in individual infection probability following exposure to clade 2.3.4.4b viruses (mean: 0.252; by virus origin: 0.25 for avian and 0.07 for bovine) compared with earlier clades (mean range: 0.05–0.42) (Fig. 4E).

## DISCUSSION

Through the collation and analyses of available data on reported human HPAI A(H5) cases reported worldwide from 1 January 1997 through 31 July 2025, we demonstrate the geographic succession of goose/Guangdong-lineage HPAI A(H5) virus clades. Our results demonstrate that individual biological, viral, and surveillance factors collectively complicate the assessment of the true disease severity of HPAI A(H5) cases. By incorporating case ascertainment into the estimation of the denominator for case hospitalization and case fatality risks, we generated robust estimates of clinical severity profiles for HPAI A(H5) cases. We show that disease severity varied considerably across HPAI A(H5) virus subtypes and clades, with comparable case fatality observed for bovine-origin HPAI A(H5N1) virus, clade 2.3.4.4b and HPAI A(H5N6) virus, clades 2.3.4x and 2.3.4.4b. Despite the complexity of estimating the epidemiological parameters of HPAI A(H5) viruses, our SIR models, which accounted for both animal-to-human and human-to-human transmission, provided a good fit to the data. Although transmissibility of HPAI A(H5) virus clades between humans remains low, animal-to-human transmission of more recent clade 2.3.4.4b viruses appears enhanced, regardless of virus subtype and animal origin, increasing additional opportunities for human infections.

Our phylogenetic analysis of available virus sequences from human cases reveals a striking pattern of regional clade succession. Specific clades of HPAI A(H5) viruses identified from human cases have dominated defined regions during distinct time periods, rather than causing simultaneous outbreaks across multiple areas, a pattern derived from phylogenetic analyses of HPAI A(H5) viruses from all hosts, as reported previously [5]. This contrasts with the relatively uniform distribution sometimes observed with viruses identified from infected animals such as clade 2.3.2.1x and 2.3.4.4b. This trend is likely because human-derived virus sequences are more likely to represent the dominant viral clades circulating in animal hosts, thereby making the clade turnover appear more punctate in humans. While we identified distinct spatiotemporal patterns in clade-specific dominance, the complex mechanisms driving clade-specific dominance in both zoonotic and rare past episodes of limited, non-sustained human-to-human transmission of any HPAI A(H5) virus are not fully elucidated. Previous studies have shown that vaccination of poultry against avian influenza A virus subtypes is directly associated with viral dynamics through immune pressure-driven selection [22]. For example, an increase in antigenic evolution of A(H5) viruses in China was observed following the implementation of the mass A(H5) poultry vaccination in 2005 and the ongoing A(H5–H7) poultry vaccination since 2017 [23]. Given the scope of our assessments, we did not conduct a quantitative comparative analysis of the association between poultry vaccination and virus evolutionary dynamics. Future studies could assess whether and to what extent widespread use of A(H5) poultry vaccination impacts HPAI A(H5) virus reassortment, evolution, and transmissibility among poultry and other animals.

Viral- and human-related factors have contributed to the region-specific epidemiology and disease severity in reported human HPAI A(H5) cases. Currently, the relative contributions of many potential factors (e.g. virus virulence, exposure routes, receptor-binding tropism, surveillance approaches, clinical management practices, underlying partial cross-protective immunity) influencing the clinical severity observed in HPAI A(H5) cases remain to be disentangled. The high prevalence of conjunctivitis among dairy workers infected with clade 2.3.4.4b viruses in the United States (83%) [16,24], along with its occurrence in a critically ill adolescent case with HPAI A(H5N1) virus, clade 2.3.4.4b infection in British Columbia [25], suggests that human infections with this clade may be more likely to be associated with conjunctivitis than infections caused by other virus clades [26]. It is also likely that the abundance of α2,3-linked sialic acid receptors on conjunctival epithelial cells enables the A(H5) virus to use the mucus membranes of the human eye as an initial site for its replication through contact transmission such as touching the eyes with virus contaminated gloves or fingers or when raw milk containing high levels of HPAI A(H5N1) virus splashes onto the faces of dairy workers [1,26–28]. Such milder illness might also be linked to the characteristics of the affected population, who were generally healthy young farm workers, and to the fact that increased active surveillance in this occupationally-exposed population following the COVID-19 pandemic enabled rapid case detection and clinical management (e.g. early access to influenza antiviral treatment) [16]. Despite a relatively low case-fatality risk of clade 2.3.4.4b viruses (0.7%), these viruses have caused severe and critical illness in patients in Canada [29], Chile [30], China [31], Ecuador [32], the United States [33,34], and Mexico [35], with some fatal outcomes.

This study has several limitations. We relied upon the available reported case data, and corresponding virus clade information for human HPAI A(H5) cases were retrospectively collected from published data. This may also introduce limitations to our transmission models in terms of both the underlying model structure and assumptions. For example, we inferred specific disease transmission parameters and case ascertainment solely from case incidence data, without incorporating additional data from active case investigations or other case detection approaches (e.g. sero-epidemiological investigations in high-risk populations). This hinders our models’ ability to account for ascertainment at a finer spatiotemporal scale and for potential variation in individual susceptibility to HPAI A(H5) viruses in the general population. Additionally, we cannot exclude the possibility that some reported human HPAI A(H5) cases may not represent true infections. Examples include asymptomatic HPAI A(H5N1) cases reported in Spain [36], a case of fatigue only in Colorado, United States [37], as well as seven reported asymptomatic HPAI A(H5N8) cases in Russia [20], all that lacked serologic testing results meeting WHO criteria for confirmed cases. Furthermore, our virus phylogenetic and epidemiological analyses were limited by the public availability of individual case data and the robustness of virologic/genomic surveillance across different settings. Given the inherent bias in global surveillance of HPAI A(H5) viruses and its unpredictable changes, largely attributable to uneven genomic sampling (with only 50% of animal outbreaks and 0.2% of cases in animals were sequenced [5]), we cannot exclude the possibility that the inferred start and end time of virus clade epidemics might be biased. Moreover, in most HPAI A(H5) cases, data on genotype, antiviral treatment or other clinical management were not available and could not be included in our analyses. Potential genotype variations in the effectiveness of and regional differences in the use of antiviral treatment are unknown and could further widen the large differences in disease severity and outcomes of HPAI A(H5) cases that we have estimated.

In conclusion, we identified frequent transitions and regional succession of HPAI A(H5) virus clades that have caused sporadic human infections, along with a clear geographic dominance of each virus clade. We provide a quantitative evidence base for monitoring epidemiological profiles and transmissibility of human HPAI A(H5) virus infections. While the risk of HPAI A(H5) virus transmission between humans remains very low to date, the zoonotic transmission potential has increased following the emergence and widespread circulation of clade 2.3.4.4b viruses, regardless of virus subtype and animal origin. As HPAI A(H5) viruses continue to evolve, including through genetic reassortment, and spread globally among birds, with spillover and evidence of HPAI A(H5N1) virus circulation among some mammals [38,39], efforts are needed to increase use of appropriate personal protective equipment and reduce human exposure to infected poultry and other animals worldwide, including to infected dairy cattle in the United States. Ongoing global surveillance of HPAI A(H5) viruses and associated human infections with standardized collection and analyses of epidemiologic, clinical, and virologic data will help inform the development and implementation of preventive measures against human infections, and pandemic influenza preparedness and response.

## MATERIALS AND METHODS

### Case definition

The definition of a confirmed HPAI A(H5) case was based on the WHO case definition for human infections with avian influenza A(H5) virus requiring notification under the International Health Regulations (2005), that is, a patient with defined clinical signs, epidemiological linkage, and laboratory confirmation by an influenza laboratory accepted by WHO [40]. In contrast, for the purposes of this analysis, a possible case refers to a person with an unexplained acute respiratory illness who is epidemiologically linked by time, place and exposure to a confirmed case or sick/dead animals, and whose infection was identified by a country or local institution but did not meet WHO criteria or was not reported to the WHO. A cluster of HPAI A(H5) cases was defined as a group of one or more confirmed cases and additional confirmed and possible cases with household contacts or shared exposure to the same infected animal population (e.g. neighbors in the same village or coworkers in the same facility), with illness onset within a 7-day period. Within a cluster, the index case referred to the case with the earliest onset data, while remaining cases in the same cluster were termed possible secondary cases.

### Data collection

#### Case line list

Human HPAI A(H5) case data were identified and compiled according to the case definitions above. The initial dataset included cases provided by national health agencies (*N* = 226) [14,41], and those compiled from WHO reports (*N* = 878) (Table S1, Fig. S1). The latter included the WHO Disease Outbreak News [42], the WHO Weekly Epidemiological Record [43], the WHO risk assessments and summaries of influenza at the human-animal interface [44], and the WHO Western Pacific Region’s Avian Influenza Weekly Update [45]. After matching with this initial case line list from national and WHO sources using case age, gender, illness onset date and location, we supplemented information for 381 cases from other publicly available sources (including FluTrackers [46], ProMED-mail [47], the Center for Infectious Disease Research and Policy [48], and the websites of ministries of health in individual countries or regions, and the Centers for Disease Control and Prevention). We also systematically searched the MEDLINE/PubMed, Embase, and Web of Science databases for articles published from 1 January 1997 through 31 July 2025. Articles containing information on individual cases (*N* = 140) were identified using the search terms ‘(H5N1 [title] OR H5N6 [title] OR H5N8 [title]) AND (patient*[title] OR person*[title] OR human*[title] OR case*[title])’, without any language restrictions (Fig. S1). After removing 89 duplicate reports identified by cross-checking case data from national/WHO sources and published articles, 1153 cases remained in the final case line list.

Individual case data were extracted by a trained data collection team and entered in a standardized database comprising three sections: demographic characteristics, key timelines (including exposure timeline, symptom onset, hospitalization, and outcome dates) and exposure history (Table S2). For each individual case’s data, a cross-check was performed by three members of the data collection team to ensure data accuracy, and first-hand data reported by local health authorities were selected when discrepancies in the reports were identified. Data on the clade of HPAI A(H5N1), A(H5N6) and A(H5N8) viruses sequenced from cases were collated from WHO or local public health reports. For individual cases without sequencing data specifying the clade, the infection was presumed to belong to the dominant HPAI A(H5N1), A(H5N6) or A(H5N8) virus clade present in birds, poultry, other animals, or human cases that occurred in the same time period and area (see details in Supplementary Text, Fig. S4).

#### Virus sequences

We retrieved HPAI A(H5) virus HA sequences from human cases and other animal hosts available in the GISAID and GenBank databases on 31 July 2025. The sequence data from the two databases were then subjected to quality control and curation. The two validated datasets, which included HPAI A(H5) virus sequences from 1997 onwards, were then merged, resulting in a final dataset of 40 988 HPAI A(H5) virus sequences (including 838 sequences from human cases; see details in Supplementary Text). For each curated sequence, we first manually determined its virus clade. To do this, we established a HPAI A(H5) virus HA gene reference database containing specific clade information. Within this HA gene reference database, up to 10 sequences per HPAI A(H5) virus clade (or all available sequences for clades with fewer than 10 entries) were downloaded from GISAID and annotated with the corresponding clade, then integrated into the above-mentioned human sequence database. The curated sequences were aligned using MAFFT v7.505, followed by removal of ambiguously aligned regions using trimAL v1.4.rev15. Finally, a maximum likelihood (ML) tree was constructed using IQ-TREE v2.1.4, and clade distributions were assigned to all virus sequences from human cases based on the reference sequence database. We additionally used our final dataset to assign virus clades with Nextclade and GISAID and cross-checked these against our manually assigned clades to assess clade assignment accuracy, yielding a final consistency of >99%.

### Statistical and phylogenetic analysis

#### Phylogenetic analysis

We analyzed the spatiotemporal distribution of HPAI A(H5) virus clades from both animal (*N* = 40 510) and human hosts (*N* = 838)—excluding any other subtypes of goose/Guangdong-lineage A(H5) viruses—across countries during three major epidemic periods of HPAI A(H5) virus infections of animal hosts and humans: in South-East Asia (1997–2003), across Africa and Asia (2004–2021) and clade 2.3.4.4b outbreaks in Asia, Europe and the Americas (2022–2025). Given the need for computational efficiency in inferring the total host phylogeny, we proposed a stratified subsampling of our final dataset above to create spatiotemporally representative sequences while maintaining genetic diversity, thereby enabling further phylogenetic analysis (see details in Supplementary Text). We realigned the subsampled sequence dataset (*N* = 7445) using MAFFT v7.505 and removed ambiguously aligned regions using trimAl v1.4.rev15. A maximum likelihood (ML) tree was constructed using IQ-TREE v2.1.4 under the best-fit nucleotide substitution model of GTR + F + I+Γ_4_ (selected based on the Bayesian Information Criterion). Temporal signal assessment and time-calibrated phylogenetic analysis were performed using TreeTime v2.4.1. Moreover, we evaluated the molecular clock signal using the root-to-tip regression analysis. The reconstruction of time-calibrated phylogenies was then performed using: (1) coalescent skyline model to infer population dynamics; (2) relaxed molecular clock dealing with rate variation across branches (clock-std-dev = 0.001); (3) a maximum of 5 iterations to ensure convergence; (4) confidence intervals for divergence time estimates; (5) polytomies resolved via maximum likelihood. The phylogenetic trees were analyzed and visualized using the ape and ggtree packages in R. Additionally, to map the evolutionary history of each virus associated with reported human cases of HPAI A(H5) onto the total host phylogeny, we built a tanglegram linking the total host phylogeny and the phylogeny of zoonotic human infections by using the Nextstrain pipeline.

#### Statistical analysis

We used information on age, sex, location of detection, exposure history, dates of symptom onset and hospital admission, and available clinical outcomes to describe the demographic and epidemiological characteristics of human HPAI A(H5) cases. We conducted statistical analyses of these characteristics by stratifying HPAI A(H5N1) cases into distinct virus clade clusters derived from phylogenetic analysis: 1.x, 2.1x and 2.1.3.2x, 2.2x and 2.2.1x, 2.3.4x, 2.3.4.4b and others. For HPAI A(H5N6) cases, we stratified them into two previously identified clade clusters: 2.3.4x and 2.3.4.4b. We then compared clade-specific differences in case sex, clinical constellation, hospitalization rate, and exposure history using Fisher exact test, χ^2^ test, or the Mann–Whitney U test, while the Kruskal–Wallis H test was used to compare age distributions. Additionally, generalized linear mixed models with a logit link function and country-level random effects were employed to identify factors associated with the reported fatality risk for confirmed cases of various HPAI A(H5) virus subtypes and clades. In this multivariable analysis, we considered three main types of factors: case biological characteristics (age and sex), virus characteristics [virus subtype, clade and animal origin (avian or bovine)] and the quality of the surveillance system. In particular, we defined different levels of disease surveillance quality in 1997–2025, which were accelerated by global responses to public health emergencies: the SARS outbreaks in 2003, the 2009 H1N1 pandemic, the Ebola outbreaks in 2014 and the COVID-19 pandemic since 2020 [49–51].

### Mathematical modelling

#### Disease severity estimates

To determine the clinical severity profile of human HPAI A(H5) virus infections, we first corrected for under-reporting and estimated the total number of symptomatic infections for each virus clade with at least 20 reported human cases in 1997–2025. Specifically, following our previously established Bayesian Poisson model for analyzing population-based time series of case onsets [21], we assumed a constant daily force of onset ${\lambda }_{i,k}$ in country *i* during its outbreak period in year *k* (i.e. the time from the first to the last case onset in that year), with a case ascertainment probability ${p}_{i,k}$ (as a comprehensive measure reflecting both health seeking biases and diagnostic testing quality). Under this assumption, the detected case counts, ${x}_{i,k,t}$, would follow a Poisson distribution with an expected mean of ${\lambda }_{i,k}{p}_{i,k}$. We jointly estimated the country- and year-specific daily forces of onset and case ascertainment probabilities using the Hamiltonian Monte Carlo method in CmdStanR, based on the time-specific number of cases observed in selected countries. We then determined clade-specific case hospitalization and fatality risks as the reported number of hospitalizations and deaths divided by our estimated total number of onsets.

#### Transmission dynamics in humans

Focusing on HPAI A(H5) virus clades that led to a substantial number of reported human cases, we fitted a discrete-time SIR model to determine their animal-to-human and human-to-human transmission potential using the reported case data in five selected countries (China, Indonesia, Egypt, the United States and Vietnam), assuming an incubation period of 3.3 days [52], and an infectious period of 9.0 days [53]. In each selected country, the number of susceptible individuals was derived from the population estimates in cities with reported cases, with the population assumed to be completely susceptible. In particular, the rates of change of each compartment are described in Equations (1)–(3):


(1) \begin{eqnarray*}
\frac{{{\rm d}S}}{{{\rm d}t}} = - \beta (t)I (t)S (t) - \lambda (t)S (t),
\end{eqnarray*}

(2) \begin{eqnarray*}
\frac{{{\rm d}I}}{{{\rm d}t}} = \beta ( t )I( t)S( t) + \lambda ( t)S( t) - \sigma I( t),
\end{eqnarray*}

(3) \begin{eqnarray*}
\frac{{{\rm d}R}}{{{\rm d}t}} = \sigma I\left( t \right),
\end{eqnarray*}

where *S, I* and *R* represent the susceptible, infected and recovered proportions of the population; $\lambda ( t )$ and $\beta ( t )$ are the time-varying transmission rates attributable to animal-to-human and human-to-human transmissions, respectively; $\sigma $ is the rate of recovery from infection. We then determined the expected number of cases at day *t* as $C( t ) = N\rho \frac{{{\rm d}I}}{{{\rm d}t}}$, where *N* is the total population size and $\rho $ denotes a constant case ascertainment probability during the study period. The model was fit using a negative binomial likelihood, based on the Hamiltonian Monte Carlo method. Three independent chains were used for the two models above, with each model having 1000 warmup samples and 2000 post-warmup samples. Convergence was assessed by visual assessment of chain mixing and by R-hat convergence diagnostic. To obtain the parameter estimates, we calculated the median of the posterior distribution for each parameter, as well as the 95% credible interval.

In addition, we assessed HPAI (H5N1) virus clade-specific human-to-human transmission potential by estimating the probability of infection upon exposure to an infected case, *p*. Here, we applied our previously established method [54,55] using data from all clustered human HPAI A(H5N1) cases with complete exposure history and household contact information. This analysis assumes an equal probability of detecting both sporadic and clustered cases. The number of human HPAI A(H5N1) cases in a household with the size *n* was modeled using a binomial distribution, *Bin*(*n, p*).

All statistical analyses were performed in R version 4.4.3.

## Supplementary Material

nwaf471_Supplemental_File

## Data Availability

Because of privacy and ethical reasons, individual case details cannot be made public, but they are available from the corresponding author (H.Y.) on reasonable request. We have provided the synthetic dataset and aggregated data generated in this study. These datasets and codes to reproduce the main results in this study are deposited in Zenodo at https://doi.org/10.5281/zenodo.17463750.

## Notes

***Conflict of interest statement.*** H.Y. has received research funding from Sanofi Pasteur, Shenzhen Sanofi Pasteur Biological Products Co., Ltd, Shanghai Roche Pharmaceutical Company, and SINOVAC Biotech Ltd. B.J.C. has consulted for AstraZeneca, Fosun Pharma, GlaxoSmithKline, Haleon, Moderna, Novavax, Pfizer, Roche, and Sanofi Pasteur. None of the research funding received is related to human HPAI A(H5N1) virus infections. The findings and conclusions in this report are those of the authors and do not necessarily represent the official position of the Centers for Disease Control and Prevention.
