# Supplementary material for: Human infections with avian influenza A(H5) viruses with potential pandemic risk: 1997–2025
Source: Natl Sci Rev. 2025 Nov 10;13(7):nwaf471. doi: 10.1093/nsr/nwaf471 (PMC13127288; doi:10.1093/nsr/nwaf471)
Supplement: nwaf471_Supplemental_File [file nwaf471_supplemental_file.docx]

**Supplementary Information**

**Table of Contents**

[Supplementary Text 2](#_Toc212717229)

[Supplementary Tables 4](#_Toc212717230)

[Table S1. WHO and other publicly available data sources for reported human HPAI A(H5) cases. 4](#_Toc212717231)

[Table S2. Variables and their completeness in the data extraction form. 9](#_Toc212717232)

[Table S3. Factors associated with case fatality using generalized linear mixed model, based on data from reported HPAI A(H5) confirmed cases only. 12](#_Toc212717233)

[Supplementary Figures 13](#_Toc212717234)

[Figure S1. Flowchart of individual case data collection. 13](#_Toc212717235)

[Figure S2. Time-scaled maximum likelihood tree of HPAI A(H5) virus HA sequences subsampling from all HPAI A(H5) virus sequences.* 14](#_Toc212717236)

[Figure S3. Tanglegram linking human A(H5) events to the total host phylogeny. 15](#_Toc212717237)

[Figure S4. Global clade turnover dynamics of HPAI A(H5) virus sequences from May 1997 to July 2025.* 16](#_Toc212717238)

[Figure S5. Temporal distribution of human HPAI A(H5) virus sequences across geographical locations.* 17](#_Toc212717239)

[Figure S6. Model-based mean daily number of human HPAI A(H5N1) virus infections by virus clade and epidemic months.* 18](#_Toc212717240)

[References 19](#_Toc212717241)

# Supplementary Text

**Collation and deduplication of genetic datasets**

To address potential duplicate HPAI A(H5) virus sequences contained in both GISAID and GenBank, we implemented our previous established de-duplication procedure within and between repositories.^1^ First, within each repository, we identified sequences with the same isolate names, sampling dates/locations and consensus sequences, retaining the sequence with the earlier collection date. Then, we cross-queried genetic sequences in both repositories and considered them as duplicates if they had: 1) the same isolate name or 2) the same sampling location, collection date, and consensus sequence. These two steps excluded a total of 2,199 (5.1 %) identified duplicate sequences.

We then performed data quality assessment for each sequence in the combined dataset using the following steps. First, given our analyses were based on the coding region of sequences, only HPAI A(H5) virus sequences covering at least 85% of the HA gene coding region (~1,700 bp in full) were retained. Second, we retained only sequences with good quality, as defined by the quality control criteria for AIV H5 Nextclade database,^2^ including quality metrics for ambiguous nucleotides, frameshifting insertions or deletions, identification of private mutations. Third, we removed any genetic data lacking complete metadata on sampling date or location.

**Subsampling of genetic dataset**

Given the need for computational efficiency in inferring the total host phylogeny, we proposed a stratified subsampling of our final dataset above to create spatiotemporally representativeness sequences while maintaining genetic diversity, thereby enabling further phylogenetic analysis. Subsampling was done by collapsing monophyletic clusters with identical spatiotemporal metadata (country for spatial scale and month for temporal scale), host type, and clade into three representative sequences. For each eligible cluster, we randomly retained three sequences and removed the remainder, thereby providing a systematic, unbiased reduction of the initial dataset. All sequences were first aligned using a multiple sequence alignment algorithm, and a maximum-likelihood tree was subsequently inferred using FastTree. Phylogenetic outliers were identified and removed through the root-to-tip regression in TempEst, after which the tree was reconstructed without them. Using the R package for Analysis of Phylogenetics and Evolution (APE), we parsed this pruned tree to delineate country-specific clusters and removed redundant sequences from each cluster. Given that the objective of this step is to reduce dataset size, we did not incorporate branch-support values into the clustering criteria, thereby avoiding the need for arbitrary threshold settings. The final subsampled dataset contained 7,445 of all 40,988 HPAI A(H5) sequences. To ensure a comprehensive reconstruction of the zoonotic transmission history, all human-derived sequences were retained in the subsample.

**Determining HPAI A(H5) virus clades**

For individual cases without laboratory results specifying the clade, the infection was presumed to belong to the dominant HPAI A(H5N1), A(H5N6) or A(H5N8) virus clade present in birds, poultry, other animals, or human cases that occurred in the same time period and area. In particular, for human HPAI A(H5) cases identified during periods when only one clade was circulating, such as those in Cambodia, Egypt, and Thailand as shown in **Fig. S4**, we directly assigned their viral clade based on the clade identified in animal hosts during the same time period. However, for HPAI A(H5N1) cases that occurred during periods involving the co-circulation of more than one virus clade, we addressed their clade attribution as follows: 1) for cases in Indonesia during 2006–2007, when virus clades 2.1x and 2.1.3.2x were co-circulating, we assigned them to the higher-level clade 2.1x; 2) for cases in southern China in 2014, we assigned them to the most prevalent virus clade 2.3.4x, which was responsible for local human HPAI A(H5N1) virus infections during that period.

# Supplementary Tables

## Table S1. WHO and other publicly available data sources for reported human HPAI A(H5) cases.

| **WHO region** | **Location** | **Subtype** | **Number of cases** | **Data Sources** |
| --- | --- | --- | --- | --- |
| **African Region** |  |  |  |  |
|  | Nigeria  (n=2) | H5N1  (N=2) | 1 confirmed case  1 possible case | 1. World Health Organization. Disease Outbreak News (DONs) - Indonesia. https://www.who.int/emergencies/disease-outbreak-news ^3^. 2. ProMED-mail. http://www.promedmail.org/ ^4^. 3. World Health Organization. Weekly Epidemiological Record (WER). https://www.who.int/publications/journals/weekly-epidemiological-record ^5^. 4. CIDRAP. Nigeria confirms its first human case of avian flu. https://www.cidrap.umn.edu/avian-influenza-bird-flu/nigeria-confirms-its-first-human-case-avian-flu ^6^. |
| **Region of the Americas** |  |  |  |  |
|  | Canada  (n=2) | H5N1  (N=2) | 2 confirmed cases | 1. World Health Organization. Risk assessments and summaries of influenza at the human-animal interface. https://www.who.int/teams/global-influenza-programme/avian-influenza/monthly-risk-assessment-summary ^7^. 2. ProMED-mail. Posts on 12 Jan 2014. <http://www.promedmail.org/> ^4^. 3. Center for Infectious Disease Research and Policy (CIDRAP). Post in on 8 Jan 2014 and 14 Jan 2014. https://www.cidrap.umn.edu/ ^8^. 4. Flu Trackers. 2016+ Global H5N1 Human Cases List. https://flutrackers.com/ ^9^. |
|  | Chile  (n=1) | H5N1  (N=1) | 1 confirmed case | 1. Flu Trackers. 2016+ Global H5N1 Human Cases List. https://flutrackers.com/ ^9^. 2. Castillo A, et al. Journal of Travel Medicine, 2023; 1–3 ^10^. |
|  | Ecuador  (n=1) | H5N1  (N=1) | 1 confirmed case | 1. World Health Organization. Human infection caused by avian influenza A(H5) - Ecuador. https://www.who.int/emergencies/disease-outbreak-news/item/2023-DON434 ^11^. 2. Flu Trackers. 2016+ Global H5N1 Human Cases List. https://flutrackers.com/ ^9^. |
|  | United States  (n=78) | H5N1  (N=78) | 71 confirmed cases  7 possible cases | 1. US CDC. H5 Bird Flu: Current Situation. https://www.cdc.gov/bird-flu/situation-summary/index.html ^12^. 2. World Health Organization. Risk assessments and summaries of influenza at the human-animal interface. https://www.who.int/teams/global-influenza-programme/avian-influenza/monthly-risk-assessment-summary ^7^. 3. 2016+ Global H5N1 Human Cases List. Flu Trackers. https://flutrackers.com/ ^9^. 4. Darg S, et al. N Engl J Med, 2024. doi: 10.1056/NEJMoa2414610 ^13^. 5. Drehoff CC, et al. MMWR Morb Mortal Wkly Rep, 2024; 73(34):734-739 ^14^. 6. Rolfes et al., Nat Med, 2025. doi: 10.1038/s41591-025-03905-2 ^15^. |
| **European Region** |  |  |  |  |
|  | Azerbaijan  (n=9) | H5N1  (N=9) | 8 confirmed cases  1 possible case | 1. World Health Organization. Weekly Epidemiological Record (WER). https://www.who.int/publications/journals/weekly-epidemiological-record ^16,17^. 2. Gilsdorf A, et al. Euro Surveillance, 2006; 11(5): 122-126 ^18^. |
|  | Russia  (N=7) | H5N8  (N=7) | 7 confirmed cases | 1. Pyankova1 OG, et al. Euro Surveill, 2021; 26(24): 2100439 ^19^.  2. World Health Organization. Disease Outbreak News (DONs) - Indonesia. https://www.who.int/emergencies/disease-outbreak-news ^20^. |
|  | Spain  (n=2) | H5N1  (N=2) | 2 confirmed cases | 1. World Health Organization. Avian Influenza A (H5N1) – Spain. https://www.who.int/emergencies/disease-outbreak-news/item/2022-DON420 ^21^. 2. Flu Trackers. 2016+ Global H5N1 Human Cases List. https://flutrackers.com/ ^9^. 3. Aznar E, et al. Euro Surveill, 2023; 28(8): 2300107 ^22^. |
|  | Turkey  (n=12) | H5N1  (N=12) | 12 confirmed cases | 1. World Health Organization. Weekly Epidemiological Record (WER). https://www.who.int/publications/journals/weekly-epidemiological-record ^5^. 2. Oner AF, et al. N Engl J Med, 2006; 355(21): 2179-2185 ^23^. 3. A. Bay et al. European Journal of Radiology, 2007; 61: 245–250 ^24^. |
|  | United Kingdom  (n=6) | H5N1  (N=6) | 6 confirmed cases | 1. World Health Organization. Avian Influenza A (H5N1) – Spain. https://www.who.int/emergencies/disease-outbreak-news/item/2022-DON420 ^21^. 2. UK Health Security Agency. Investigation into the risk to human health of avian influenza (influenza A H5N1) in England: technical briefing 5. https://www.gov.uk/government/publications/avian-influenza-influenza-a-h5n1-technical-briefings/investigation-into-the-risk-to-human-health-of-avian-influenza-influenza-a-h5n1-in-england-technical-briefing-5 ^25^. 3. Flu Trackers. 2016+ Global H5N1 Human Cases List. https://flutrackers.com/ ^9^. 4. CIDRAP. UK reports H5N1 in a poultry worker. https://www.cidrap.umn.edu/ ^26^. 5. Oliver I, et al. Euro Surveill, 2022; 27(5): 2200061 10/30/25 11:49:00 AM. 6. Kmietowicz Z. BMJ, 2025; 388: 1756-1833 ^27^. |
| **Eastern Mediterranean Region** |  |  |  |  |
|  | Djibouti  (n=1) | H5N1  (N=1) | 1 confirmed case | 1. World Health Organization. Avian influenza - situation in Djibouti. https://www.who.int/emergencies/disease-outbreak-news/item/2006_05_12-en ^28^. |
|  | Egypt  (n=381) | H5N1  (N=381) | 359 confirmed cases  22 possible cases | 1. World Health Organization. Risk assessments and summaries of influenza at the human-animal interface. https://www.who.int/teams/global-influenza-programme/avian-influenza/monthly-risk-assessment-summary ^7^. 2. World Health Organization. Disease Outbreak News (DONs) - Egypt. https://www.who.int/emergencies/disease-outbreak-news ^3^. 3. World Health Organization. Weekly Epidemiological Record (WER). https://www.who.int/publications/journals/weekly-epidemiological-record ^5^. 4. World Health Organization. Avian influenza A(H5N1) update in Egypt. https://www.emro.who.int/health-topics/avian-influenza/regional-situation-update.html ^29^. 5. ProMED-mail. http://www.promedmail.org/ ^4^. 6. Kandeel A, et al. Emerg Infect Dis, 2010; 16(7): 1101-1107 ^30^. |
|  | Iraq  (n=3) | H5N1  (N=3) | 3 confirmed cases | 1. World Health Organization. Risk assessments and summaries of influenza at the human-animal interface. https://www.who.int/teams/global-influenza-programme/avian-influenza/monthly-risk-assessment-summary ^7^. 2. World Health Organization. Avian influenza – situation in Iraq – Update. https://www.who.int/emergencies/disease-outbreak-news ^31^. 3. World Health Organization. Weekly Epidemiological Record (WER). https://www.who.int/publications/journals/weekly-epidemiological-record ^5^. |
|  | Pakistan (n=4) | H5N1  (N=4) | 3 confirmed cases  1 possible case | 1. World Health Organization. Weekly Epidemiological Record, 2008, vol. 83, 40. https://iris.who.int/handle/10665/241213 ^32^. 2. WHO. Disease Outbreak News - Pakistan. https://www.who.int/emergencies/disease-outbreak-news/item/2007_12_27-en ^33^. |
| **South-East Asia Region** |  |  |  |  |
|  | Bangladesh  (n=12) | H5N1  (N=12) | 12 confirmed cases | 1. World Health Organization. Risk assessments and summaries of influenza at the human-animal interface. https://www.who.int/teams/global-influenza-programme/avian-influenza/monthly-risk-assessment-summary ^7^. 2. ProMED-mail. http://www.promedmail.org/ ^4^. 3. Brooks WA, et al. Emerg Infect Dis, 2009; 15(8): 1311-1313 ^34^. 4. World Health Organization. Avian influenza weekly update 2025. https://iris.who.int/handle/10665/380024 ^35^. |
|  | India  (n=2) | H5N1  (N=2) | 2 confirmed case | 1. World Health Organization. Human infection with avian influenza A(H5N1) ｰ India. https://www.who.int/emergencies/disease-outbreak-news/item/human-infection-with-avian-influenza-a(h5n1)-%EF%BD%B0-india ^36^. 2. Flu Trackers. 2016+ Global H5N1 Human Cases List. https://flutrackers.com/ ^9^. |
|  | Indonesia  (n=211) | H5N1  (N=211) | 200 confirmed cases  11 possible cases | 1. World Health Organization. Risk assessments and summaries of influenza at the human-animal interface. https://www.who.int/teams/global-influenza-programme/avian-influenza/monthly-risk-assessment-summary ^7^. 2. World Health Organization. Weekly Epidemiological Record (WER). https://www.who.int/publications/journals/weekly-epidemiological-record ^5^. 3. World Health Organization. Disease Outbreak News (DONs) - Indonesia. https://www.who.int/emergencies/disease-outbreak-news ^3^. 4. ProMED-mail. http://www.promedmail.org/ ^4^. 5. Kandun IN, et al. N Engl J Med, 2006; 355(21): 2186-2194 ^37^. 6. Yang Y, et al. Emerg Infect Dis, 2007; 13(9): 1348-1353 ^38^. 7. Olsen SJ, et al. Emerg Infect Dis, 2005; 11(11): 1799-1801 ^39^. |
|  | Myanmar  (n=1) | H5N1  (N=1) | 1 confirmed case | 1. World Health Organization. Weekly Epidemiological Record (WER). https://www.who.int/publications/journals/weekly-epidemiological-record ^5^. 2. WHO Western Pacific Region. Avian Influenza Weekly Update. https://www.who.int/westernpacific/wpro-emergencies/surveillance/avian-influenza ^40^. |
|  | Nepal  (n=1) | H5N1  (N=1) | 1 confirmed case | 1. WHO. Information on Avian Influenza A (H5N1) Identified in Human in Nepal. https://www.who.int/nepal/news/detail/01-05-2019-information-on-avian-influenza-a-(h5n1)-identified-in-human-in-nepal ^41^. |
|  | Thailand  (n=28) | H5N1  (N=28) | 25 confirmed cases  3 possible cases | 1. World Health Organization. Weekly Epidemiological Record (WER). https://www.who.int/publications/journals/weekly-epidemiological-record ^5^. 2. World Health Organization. Weekly Epidemiological Record (WER). https://www.who.int/publications/journals/weekly-epidemiological-record ^5^. 3. Ungchusak K, et al. N Engl J Med, 2005; 352(4): 333-340 ^42^. 4. Olsen SJ, et al. Emerg Infect Dis, 2005; 11(11): 1799-801 ^39^. |
| **Western Pacific Region** |  |  |  |  |
|  | Australia  (n=1) | H5N1  (N=1) | 1 confirmed case | 1. World Health Organization. Risk assessments and summaries of influenza at the human-animal interface. https://www.who.int/teams/global-influenza-programme/avian-influenza/monthly-risk-assessment-summary ^7^. 2. Flu Trackers. 2016+ Global H5N1 Human Cases List. https://flutrackers.com/ ^9^. |
|  | Cambodia  (n=88) | H5N1  (N=88) | 86 confirmed cases  2 possible cases | 1. World Health Organization. Disease Outbreak News (DONs) - Combodia. https://www.who.int/emergencies/disease-outbreak-news ^3^. 2. World Health Organization. Risk assessments and summaries of influenza at the human-animal interface. https://www.who.int/teams/global-influenza-programme/avian-influenza/monthly-risk-assessment-summary ^7^. 3. World Health Organization. Weekly Epidemiological Record (WER). https://www.who.int/publications/journals/weekly-epidemiological-record ^5^. 4. Flu Trackers. Cambodia H5N1 Case Lists. https://flutrackers.com/forum/forum/cambodia/cambodia-h5n1-tracking ^43^. 5. Flu Trackers. 2016+ Global H5N1 Human Cases List. https://flutrackers.com/ ^9^. 6. Olsen SJ, et al. Emerg Infect Dis, 2005; 11(11): 1799-1801 ^39^. |
|  | Chinse mainland (n=145) | H5N1  (N=58) | 56 confirmed cases  2 possible cases | 1. Lai S, et al. Lancet Infectious Diseases, 2016; 16(7): e108-e118 ^44^. 2. World Health Organization. Risk assessments and summaries of influenza at the human-animal interface. https://www.who.int/teams/global-influenza-programme/avian-influenza/monthly-risk-assessment-summary ^7^. 3. Yu H, et al. Lancet, 2006; 367(9504): 84 ^45^. 4. Wang H, et al. Lancet, 2008; 371(9622): 1427-1434 ^46^. 5. Flu Trackers. 2016+ Global H5N1 Human Cases List. https://flutrackers.com/ ^9^. |
|  |  | H5N6  (N=87) | 87 confirmed cases | 1. World Health Organization. Avian influenza weekly update 2025. https://iris.who.int/handle/10665/380024 ^35^.   2. Centre for Health Protection, Hong Kong SAR. Avian Influenza Report. https://www.chp.gov.hk/sc/resources/29/332.html ^47^.  3. Flu Trackers. Global H5N6 Cumulative Case List. https://flutrackers.com/forum/forum/china-h5n1-h5n8-h5n6-h5n3-h5n2-h10n8-outbreak-tracking/723926-flutrackers-global-h5n6-cumulative-case-list ^48^. |
|  | Hong Kong SAR, China  (n=23) | H5N1  (N=23) | 20 confirmed cases  3 possible cases | 1. Centre for Health Protection, Hong Kong SAR. Avian Influenza Report. https://www.chp.gov.hk/sc/resources/29/332.html ^47^. 2. Center for Disease Control and Prevention. MMWR Morb Mortal Wkly Rep; 1997;46:1204-1207 ^49^. 3. Center for Disease Control and Prevention. MMWR Morb Mortal Wkly Rep; 1998;46:1245-1247 ^50^. 4. World Health Organization. Weekly Epidemiological Record (WER). https://www.who.int/publications/journals/weekly-epidemiological-record ^5^. 5. World Health Organization. Risk assessments and summaries of influenza at the human-animal interface. https://www.who.int/teams/global-influenza-programme/avian-influenza/monthly-risk-assessment-summary ^7^. 6. ProMED-mail. http://www.promedmail.org/ ^4^. 7. Peiris JS, et al. Lancet, 2004; 363(9409): 617-619 ^51^. 8. Yuen KY et al. Lancet, 1998; 351: 467–71 ^52^.10/30/25 11:49:00 AM |
|  | Laos  (n=4) | H5N1  (N=3) | 3 confirmed cases | 1. World Health Organization. Weekly Epidemiological Record (WER). https://www.who.int/publications/journals/weekly-epidemiological-record ^5^. 2. WHO Western Pacific Region. Avian Influenza Weekly Update. https://www.who.int/westernpacific/wpro-emergencies/surveillance/avian-influenza ^40^. 3. Flu Trackers. 2016+ Global H5N1 Human Cases List. https://flutrackers.com/ ^9^. 4. Puthavathana P, et al. Emerg Infect Dis, 2009; 15(1): 127-128 ^53^. |
|  |  | H5N6  (N=1) | 1 confirmed case | 1. Flu Trackers. Global H5N6 Cumulative Case List. https://flutrackers.com/forum/forum/china-h5n1-h5n8-h5n6-h5n3-h5n2-h10n8-outbreak-tracking/723926-flutrackers-global-h5n6-cumulative-case-list ^48^.  2. Bounthanom S, et al. Influenza and Other Respiratory Viruses, 2022; 16(2): 181-185 ^54^. |
|  | Viet Nam  (n=140) | H5N1  (N=140) | 131 confirmed cases  9 possible cases | 1. World Health Organization. Risk assessments and summaries of influenza at the human-animal interface. https://www.who.int/teams/global-influenza-programme/avian-influenza/monthly-risk-assessment-summary ^7^. 2. WHO Western Pacific Region. Avian Influenza Weekly Update. https://www.who.int/westernpacific/wpro-emergencies/surveillance/avian-influenza ^40^. 3. World Health Organization. Avian Influenza A(H5N1) – Viet Nam. https://www.who.int/emergencies/disease-outbreak-news ^55^. 4. Flu Trackers. Vietnam H5N1 Case Lists. https://flutrackers.com/forum/forum/asia/h5n1-h5n8-h1n08-tracking/vietnam-aa ^56^. 5. ProMED-mail. http://www.promedmail.org/ ^4^. 6. Tran TH, et al. N Engl J Med, 2004; 350(12): 1179-1188 ^57^. 7. Menno D, et al. N Engl J Med, 2005; 352(7): 686-691 ^58^. 8. Flu Trackers. 2016+ Global H5N1 Human Cases List. https://flutrackers.com/ ^9^. |

## Table S2. Variables and their completeness in the data extraction form.

| **Sections** | **Variables** | **Definition/details** | **Type** | **Format** | **Data completeness^a^** | | |
| --- | --- | --- | --- | --- | --- | --- | --- |
|  |  |  |  |  | **All**  **(n=1,104)** | **Health agencies**  **(n=226)** | **Other data sources (N=878)** |
| **I. Demographic characterisitcs** | **fid** | A unique number for identifying case in the dataset | Integer | 1 - 9999 | 100% | 100% | 100% |
|  | **diagtype** | Confirmed case or possible case (see case definition) | Integer | 1 = Confirmed case, 2 = Possible case | 100% | 100% | 100% |
|  | **country** | Name of the country where case was reported. | String |  | 100% | 100% | 100% |
|  | **ISO3** | ISO 3166-1 alpha-3 codes of each country or region | String | Three-letter country codes to represent countries, dependent territories, and special areas of geographical interest. | 100% | 100% | 100% |
|  | **WHO_region** | WHO regions where case was reported | String | Six regions: African Region, Eastern Mediterranean Region, European Region, Region of the Americas, South-East Asia Region, and Western Pacific Region | 100% | 100% | 100% |
|  | **province** | Province (administrative level 1) where the case was reported in a country. | String |  | 96.7% | 94.4% | 97.2% |
|  | **city** | Prefecture or city (administrative level 2) where the case was reported in a country. | String |  | 55.5% | 88.9% | 46.9% |
|  | **latitude** | Latitude in the geographic coordinate of the most detailed location in variables of province, city, and county. | Double | Ranging from -90 (South) to 90 (North) | 100% | 100% | 90.2% |
|  | **longitude** | Longitude in the geographic coordinate of the most detailed location in variables of province, city, and county. | Double | Ranging from -180 (West) to 180 (East) | 100% | 100% | 90.2% |
|  | **age** | Age (years) of the case when illness onset. | Double | Years. Empty = Unknown | 90.5% | 99.1% | 88.6% |
|  | **sex** | Gender of case | Integer | 1 = Male, 2 = Female, Empty = Unknown | 94.6% | 100% | 93.6% |
|  | **occupation** | Occupation of the case | String | Empty = Unknown | 17.4% | 29.2% | 13.0% |
| **II. Key timelines** | **report_date** | Date of reporting case | Date | mm-dd-yyyy, Empty = Unknown | 67.4% | 71.3% | 65.9% |
|  | **onset_date** | Date of illness onset | Date | mm-dd-yyyy, Empty = Unknown | 82.2% | 92.1% | 80.2% |
|  | **onset_year** | Year of illness onset | Integer | E.g. 2000. Empty = Unknown | 96.3% | 100% | 96.5% |
|  | **onset_month** | Month of illness onset | Integer |  | 94.7% | 100% | 94.0% |
|  | **onset_season** | Season of illness onset | Integer |  | 91.3% | 100% | 89.8% |
|  | **onset_week** | Week of illness onset | Integer |  | 91.4% | 99.1% | 82.9% |
|  | **hospitalization** | Admitted to hospital | Integer | 0 = No, 1 = Yes, Empty = Unknown | 89.5% | 95.8% | 88.2% |
|  | **hosp_date** | Date of hospital admission | Date | mm-dd-yyyy, Empty = Unknown | 70.0% | 79.2% | 68.3% |
|  | **outcome** | Final outcome of the case | Integer | 0 = death, 1 = survive, Empty = Unknown | 98.0% | 98.6% | 97.8% |
|  | **outcome_date** | Date of death or discharge (recovery, survive) | Date | mm-dd-yyyy, Empty = Unknown | 54.4% | 82.9% | 48.0% |
|  | **whe_symp** | laboratory-confirmed cases with symptoms | Integer | 0 = No, 1 = Yes, Empty = Unknown | 21.6% | 29.2% | 18.8% |
| **III. Exposure history** | **exposure** | The history of exposure | Integer | 0 = No, 1 = Yes, Empty = Unknown | 88.1% | 90.3% | 87.4% |
|  | **any_expo_am** | Any exposure to animals (including domestic poultry, wild birds and diary cattle) | Integer | 0 = No, 1 = Yes, Empty = Unknown | 85.7% | 88.4% | 84.9% |
|  | **infect_am** | Exposure to infected or potentially infected animal (including domestic poultry, wild birds and diary cattle) | Integer | 0 = No, 1 = Yes, Empty = Unknown | 57.7% | 59.3% | 54.2% |
|  | **visit_market** | Visiting live poultry/bird market | Integer | 0 = No, 1 = Yes, Empty = Unknown | 41.1% | 58.3% | 36.1% |
|  | **visit_farm** | Exposure to farms feeding poultry and livestocks | Integer | 0 = No, 1 = Yes, Empty = Unknown | 12.4% | 7.9% | 12.0% |
|  | **backyard_av** | Exposure to backyard poultry flock | Integer | 0 = No, 1 = Yes, Empty = Unknown | 41.4% | 59.3% | 36.2% |
|  | **contact_case** | Exposure to virus through case contact | Integer | 0 = No, 1 = Yes, Empty = Unknown | 83.0% | 90.7% | 80.9% |
|  | **cluster** | Belongs to a cluster with a confirmed epidemiological link | Integer | 0 = No, 1 = Yes, Empty = Unknown | 100% | 100% | 100% |
|  | **cluster_id** | Identifying number of the cluster that the case belongs to | Integer | 1 - 999 | 100% | 100% | 100% |
|  | **indexcase** | Whether the case is an index case of a cluster (the first case with the earliest onset date), if the case belongs to a cluster. | Integer | 0 = No, 1 = Yes, Empty = Unknown | 99% of cluster cases | 100% of cluster cases | 98.7% of cluster cases |
|  | **Relation** | The relationship with the cluster index case. | String |  | 95% of cluster cases | 100% of cluster cases | 93.6% of cluster cases |
|  | **Blood_relation** | Refers to a blood relationship of the possible secondary case with the cluster index case. | Integer | 0 = spouse, healthcare worker, or other unrelated family member. 1 = parent/offspring, sibling, grandparent/grandchild, uncle/aunt, or niece/nephew.  Empty = Unknown | 96.6% of cluster cases | 100% of cluster cases | 95.5% of cluster cases |

^a^ Note that there was no significant difference in data completeness between case data provided by national health agencies and that obtained from other sources (p-value = 0.282).

## Table S3. Factors associated with case fatality using generalized linear mixed model, based on data from reported HPAI A(H5) confirmed cases only.

| **Characteristics** | **Univariable analysis** | | **Multivariable analysis** | |
| --- | --- | --- | --- | --- |
|  | **OR (95%CI)** | **P-value** | **OR (95%CI)** | **P-value** |
| Age |  |  |  |  |
| 0-17 years | 0.43 (0.31, 0.6) | **5.33e-07** | 0.44 (0.32, 0.61) | **7.53e-07** |
| 18-64 years | Reference | - | Reference | - |
| ≥65 | 0.77 (0.31, 1.93) | 0.574 | 1.16 (0.4, 3.31) | 0.788 |
| Gender |  |  |  |  |
| Male | Reference | - | Reference | - |
| Female | 1.47 (1.09, 1.98) | **0.012** | 1.41 (1.03, 1.93) | **0.030** |
| Neuraminidase subtype |  |  |  |  |
| N1 | Reference | - | Reference | - |
| N6 | 0.45 (0.21, 0.96) | **0.037** | 6.30 (2.36, 16.84) | **2.39e-04** |
| Viral clade |  |  |  |  |
| 1.x | 5.62 (1.34, 23.62) | **0.018** | 11.93 (3.80, 37.47) | **2.17e-05** |
| 2.1x | 20.28 (3.89, 105.66) | **3.53e-04** | 37.80 (11.67, 122.42) | **1.38e-09** |
| 2.1.3.2x | 47.49 (5.52, 408.74) | **4.40e-04** | 104.89 (16.9, 651.17) | **5.89e-07** |
| 2.2.1x | 2.13 (0.50, 9.03) | 0.306 | 4.06 (1.37, 12.05) | **0.012** |
| 2.3.4x | 5.93 (1.67, 21.12) | **0.006** | 9.86 (3.25, 29.89) | **5.22e-05** |
| 2.3.4.4b |  |  |  |  |
| Avian origin | 0.63 (0.19, 2.08) | 0.448 | 0.37 (0.12, 1.15) | 0.087 |
| Bovine origin | Reference | - | Reference | - |
| Quality of surveillance system |  |  |  |  |
| Low (1997-2003) | 26.69 (2.82, 252.96) | **4.21e-03** | 9.39 (1.04, 84.73) | **0.046** |
| Moderate (2004-2014)^*^ | 6.87 (3.16, 14.94) | **1.10e-06** | 1.83 (1.16, 2.89) | **0.009** |
| High (2015-2019)^*^ | 5.26 (2.36, 11.73) | **5.00e-05** | - | - |
| Very high (2020-2025) | Reference | - | Reference | - |

*To ensure sufficient sample size, the two groups were merged in the multivariable analysis.

# Supplementary Figures


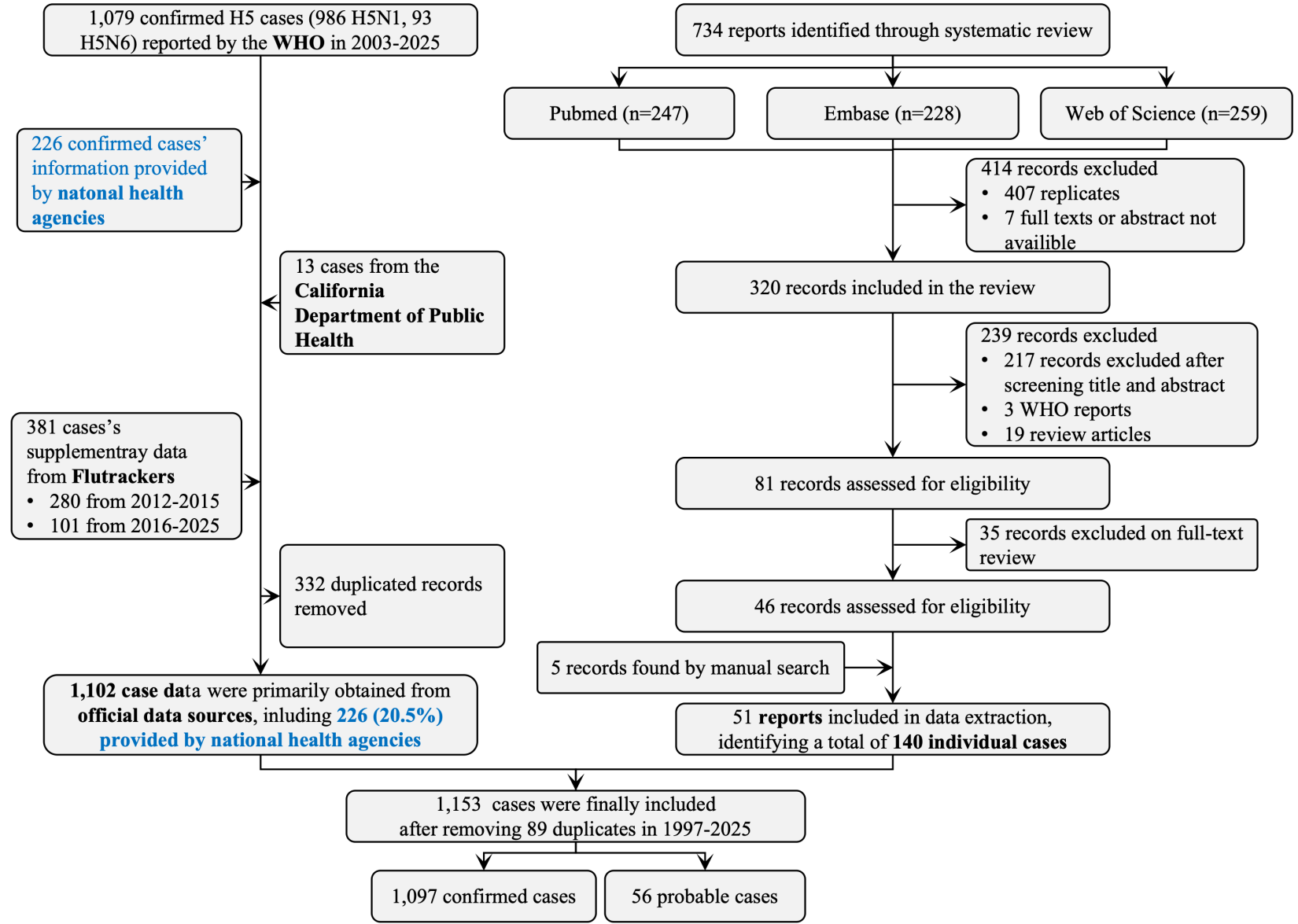


## Figure S1. Flowchart of individual case data collection.

Note that the blue text indicates individual case data provided by national health agencies.


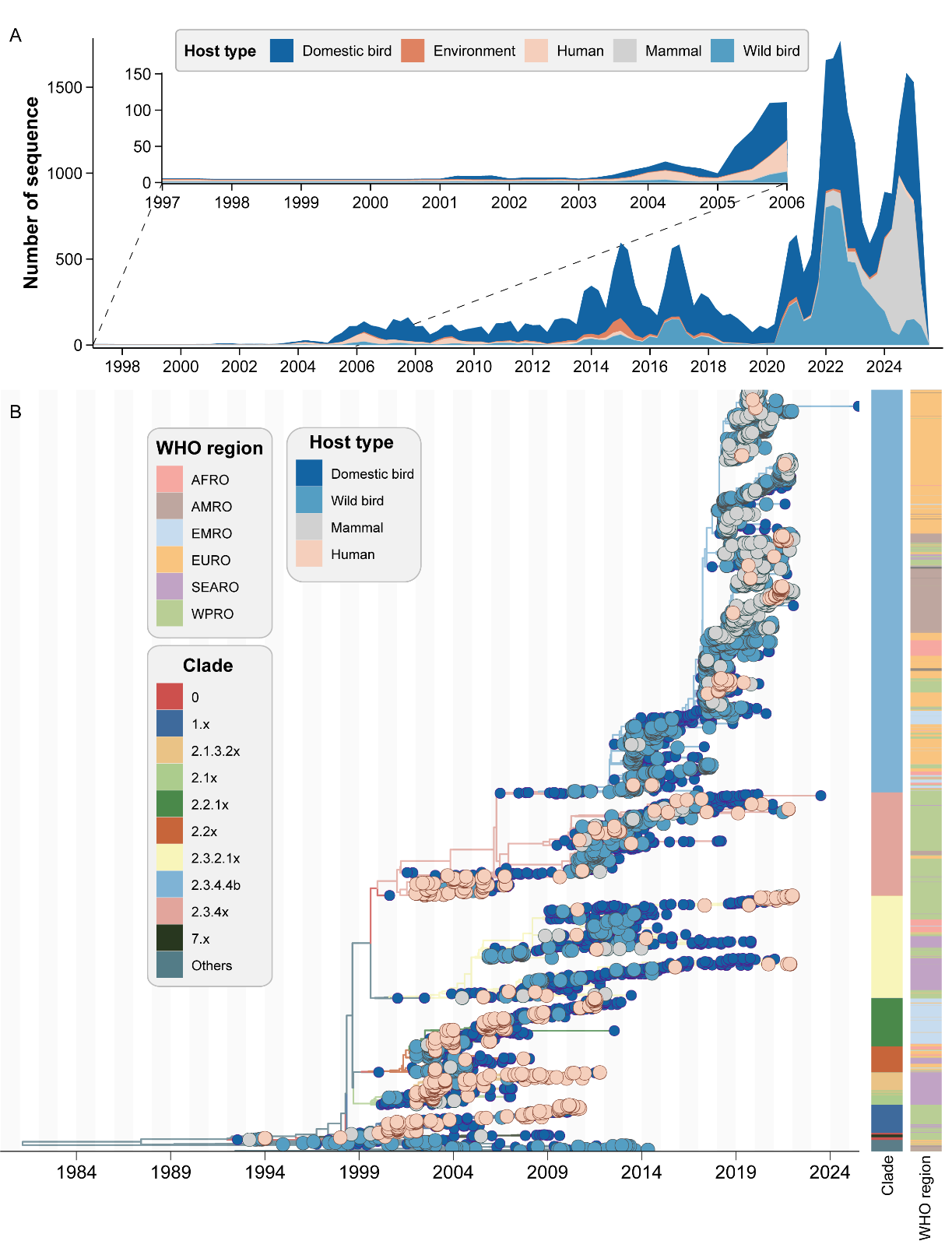


## Figure S2. Time-scaled maximum likelihood tree of HPAI A(H5) virus HA sequences subsampling from all HPAI A(H5) virus sequences.*

(**A**) Number of HA gene sequences of HPAI A(H5N1), A(H5N6) and A(H5N8) viruses (hereafter referred to as A(H5)), stratified by host type: domestic birds, wild birds, mammals, environmental samples, and humans. The stacked area shows changes in detected number of sequencies by host species over time; inset panel provide a magnified view for the period of 1997–2006. (**B**) Time-calibrated maximum likelihood phylogenetic tree constructed using HPAI A(H5) virus HA gene sequences (N= 7,445). Terminal branch points denote host species and the tip length denotes the clade information. Colored bars on the right indicate clade designations and geographical distribution of viral sequences.

*HPAI=highly pathogenic avian influenza; HA=hemagglutinin


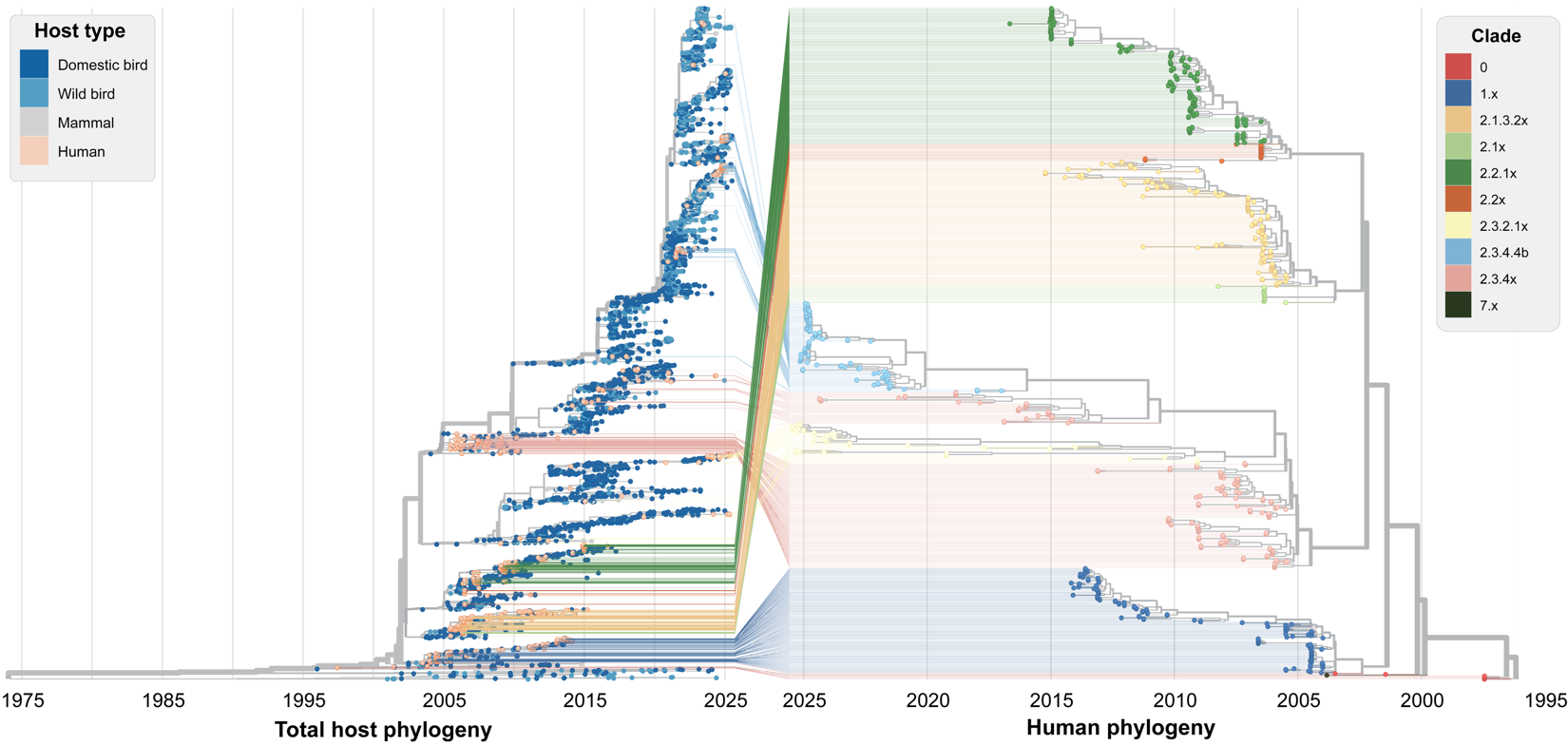


## Figure S3. Tanglegram linking human A(H5) events to the total host phylogeny.

**Left**: Time‑resolved HA phylogeny of 7,445 subsampled sequences from all host species, with tip colors denoting host type. **Right**: Time‑resolved HA phylogeny of 838 human HPAI H5 isolates, with colored tip shapes indicating major clades. Lines connect each human isolate in the right tree to its exact phylogenetic placement within the broader host phylogeny on the left, illustrating that every human spillover event follows circulation of the same virus clade in animals.


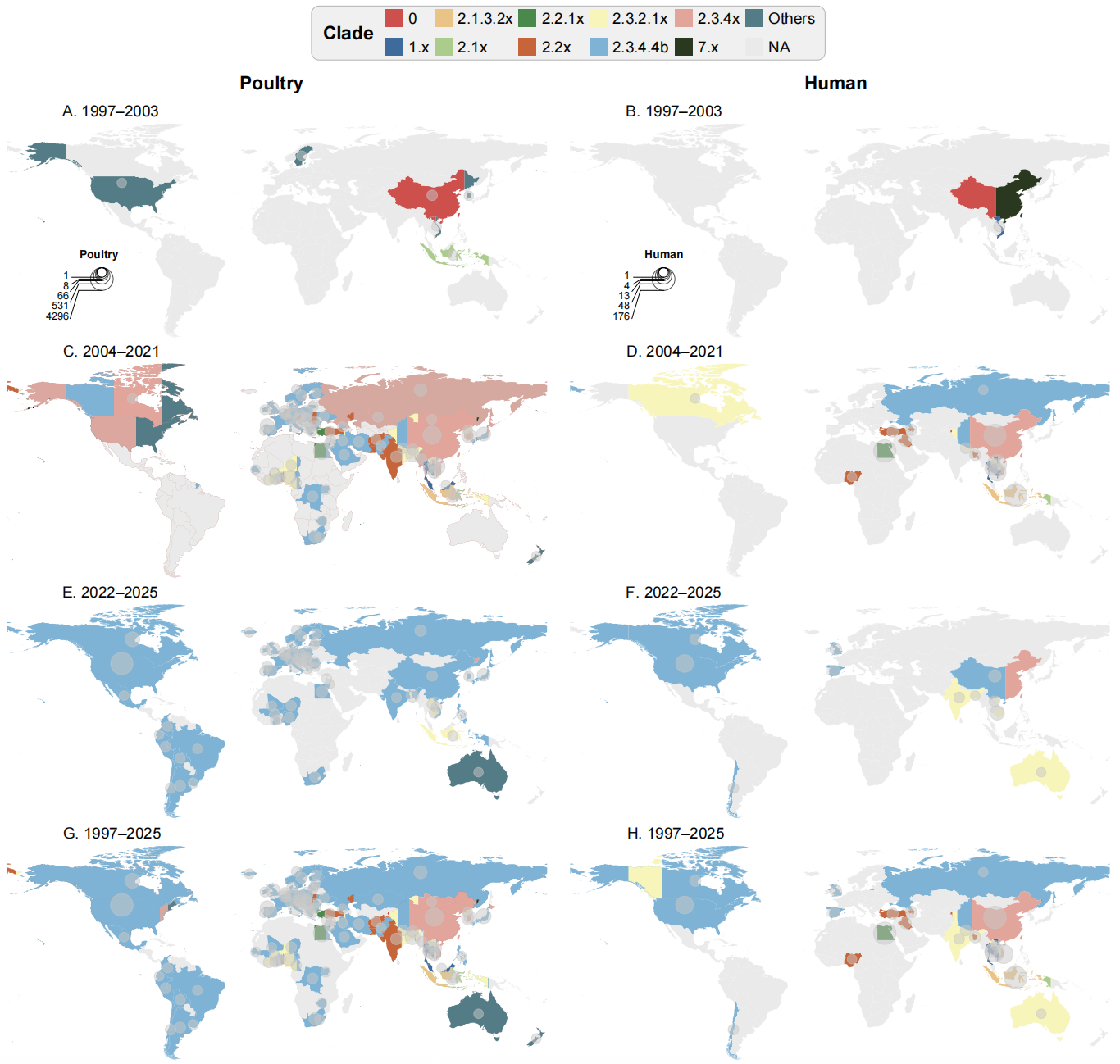


## Figure S4. Global clade turnover dynamics of HPAI A(H5) virus sequences from May 1997 to July 2025.*

The distribution of sequences from different virus clades is shown across three key HPAI A(H5) virus epidemic waves in poultry (**left**) and human (**right**): (**A-B**) 1997-2003, (**C-D**) 2004-2021, (**E-F**) 2022-present, and (**G-H**) global summaries in 1997-2025. Within each location, the proportional distribution of different virus clades is presented by the horizontal span of their respective colors. The size of grey bubble was proportional to the total count of available human HPAI A(H5) virus sequences.

*HPAI=highly pathogenic avian influenza


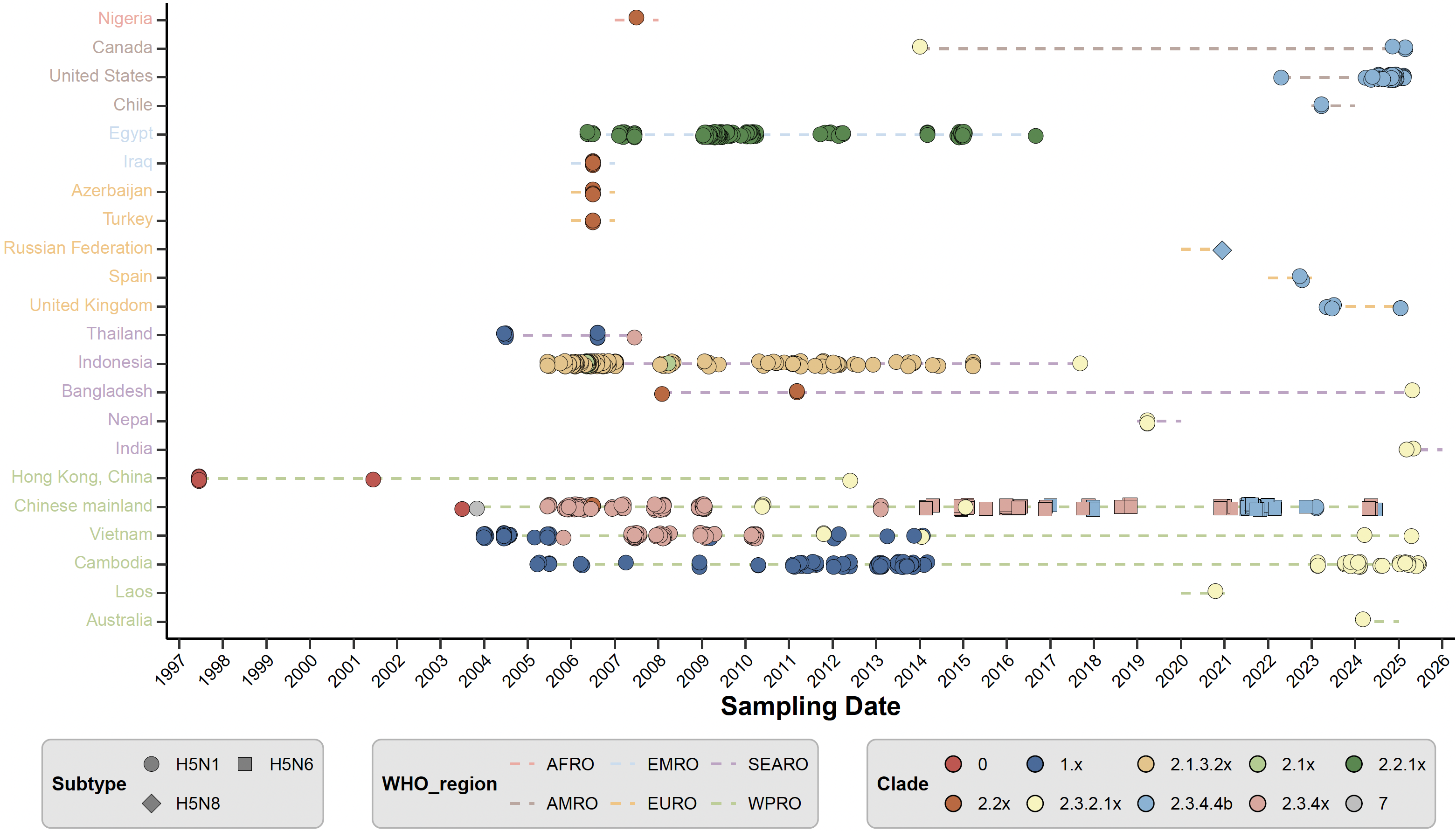


## Figure S5. Temporal distribution of human HPAI A(H5) virus sequences across geographical locations.*

Note that locations are ordered by the date of their first reported human case. Colored dashed lines and text indicate WHO regions, with line lengths representing the time span from the first to the last reported sequence in each location. Each point, square and diamond refer to individual sequences of different HPAI A(H5) virus subtypes.

*HPAI=highly pathogenic avian influenza


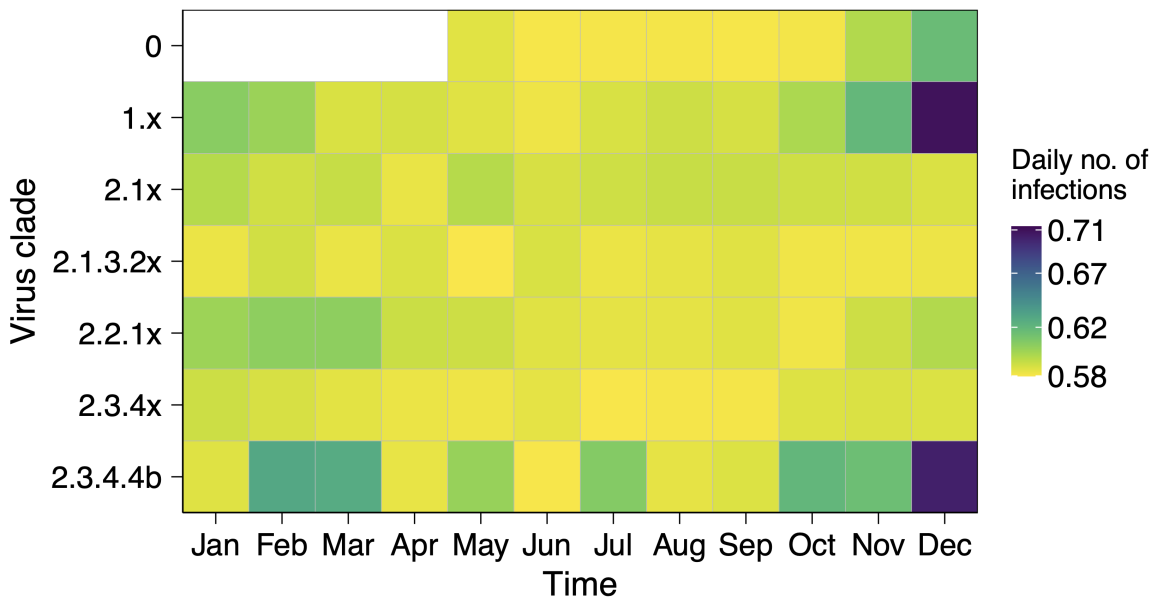


## Figure S6. Model-based mean daily number of human HPAI A(H5N1) virus infections by virus clade and epidemic months.*

Note that only the bovine-original A(H5N1) virus, clade 2.3.4.4b was included in the analyses.

*HPAI=highly pathogenic avian influenza

# References

1. Chen, Z. *et al.* COVID-19 pandemic interventions reshaped the global dispersal of seasonal influenza viruses. *Science* (2024) doi:10.1126/science.adq3003.

2. Ort, J. T., Zolnoski, S. A., Lam, T. T.-Y., Neher, R. & Moncla, L. H. Development of avian influenza A(H5) virus datasets for Nextclade enables rapid and accurate clade assignment. *bioRxiv* 2025.01.07.631789 (2025) doi:10.1101/2025.01.07.631789.

3. World Health Organization. Disease Outbreak News. https://www.who.int/emergencies/disease-outbreak-news.

4. ProMED-mail. ProMED. https://promedmail.org/.

5. World Health Organization. The Weekly Epidemiological Record (WER). https://www.who.int/publications/journals/weekly-epidemiological-record.

6. CIDRAP. Nigeria confirms its first human case of avian flu. https://www.cidrap.umn.edu/avian-influenza-bird-flu/nigeria-confirms-its-first-human-case-avian-flu (2007).

7. World Health Organization. Risk assessments and summaries of influenza at the human-animal interface. https://www.who.int/teams/global-influenza-programme/avian-influenza/monthly-risk-assessment-summary.

8. CIDRAP. CIDRAP. https://www.cidrap.umn.edu/ (2025).

9. FluTrackers. FluTrackers 2016+ Global H5N1 Human Cases List. *FluTrackers News and Information* https://flutrackers.com/forum/forum/flutrackers-high-pathogenic-h5n1-h1n08-h5n8-h5n6-h5n3-tracking-outbreaks-spread/749462-flutrackers-2016-global-h5n1-human-cases-list (2016).

10. Castillo, A. *et al.* The first case of human infection with H5N1 avian Influenza A virus in Chile. *J Travel Med* **30**, (2023).

11. World Health Organization. Human infection caused by avian influenza A(H5) - Ecuador. https://www.who.int/emergencies/disease-outbreak-news/item/2023-DON434.

12. CDC. H5 Bird Flu: Current Situation. *Avian Influenza (Bird Flu)* https://www.cdc.gov/bird-flu/situation-summary/index.html (2025).

13. Garg, S. *et al.* Highly Pathogenic Avian Influenza A(H5N1) Virus Infections in Humans. *N Engl J Med* (2024) doi:10.1056/NEJMoa2414610.

14. Drehoff, C. C. Cluster of Influenza A(H5) Cases Associated with Poultry Exposure at Two Facilities — Colorado, July 2024. *MMWR Morb Mortal Wkly Rep* **73**, (2024).

15. Rolfes, M. A. *et al.* Human infections with highly pathogenic avian influenza A(H5N1) viruses in the United States from March 2024 to May 2025. *Nat Med* 1–1 (2025) doi:10.1038/s41591-025-03905-2.

16. World Health Organization. Weekly Epidemiological Record, 2006, vol. 81, 18 [full issue]. *Weekly Epidemiological Record = Relevé épidémiologique hebdomadaire* https://iris.who.int/handle/10665/233085 (2006).

17. World Health Organization. Weekly Epidemiological Record, 2006, vol. 81, 12 [full issue]. *Weekly Epidemiological Record = Relevé épidémiologique hebdomadaire* https://iris.who.int/handle/10665/233051 (2006).

18. Gilsdorf, A. *et al.* Two clusters of human infection with influenza A/H5N1 virus in the Republic of Azerbaijan, February–March 2006. *Eurosurveillance* **11**, 3–4 (2006).

19. Pyankova, O. G. *et al.* Isolation of clade 2.3.4.4b A(H5N8), a highly pathogenic avian influenza virus, from a worker during an outbreak on a poultry farm, Russia, December 2020. *Euro Surveill* **26**, 2100439 (2021).

20. World Health Organization. Human infection with avian influenza A (H5N8) - Russian Federation. https://www.who.int/emergencies/disease-outbreak-news/item/2021-DON313.

21. World Health Organization. Avian Influenza A (H5N1) – Spain. https://www.who.int/emergencies/disease-outbreak-news/item/2022-DON420.

22. Aznar, E. *et al.* Influenza A(H5N1) detection in two asymptomatic poultry farm workers in Spain, September to October 2022: suspected environmental contamination. *Eurosurveillance* **28**, (2023).

23. Oner, A. F. *et al.* Avian influenza A (H5N1) infection in eastern Turkey in 2006. *N Engl J Med* **355**, 2179–2185 (2006).

24. Bay, A. *et al.* Radiological and clinical course of pneumonia in patients with avian influenza H5N1. *Eur J Radiol* **61**, 245–250 (2007).

25. Investigation into the risk to human health of avian influenza (influenza A H5N1) in England: technical briefing 5. *GOV.UK* https://www.gov.uk/government/publications/avian-influenza-influenza-a-h5n1-technical-briefings/investigation-into-the-risk-to-human-health-of-avian-influenza-influenza-a-h5n1-in-england-technical-briefing-5.

26. CIDRAP. UK reports H5N1 in a poultry worker. https://www.cidrap.umn.edu/avian-influenza-bird-flu/uk-reports-h5n1-poultry-worker (2025).

27. Kmietowicz, Z. Human case of avian flu detected in England. *BMJ* **388**, r177 (2025).

28. World Health Organization. Avian influenza – situation in Djibouti. https://www.who.int/emergencies/disease-outbreak-news/item/2006_05_12-en.

29. World Health Organization. Avian influenza A(H5N1) situation update, Egypt. *World Health Organization - Regional Office for the Eastern Mediterranean* http://www.emro.who.int/health-topics/avian-influenza/regional-situation-update.html.

30. Kandeel, A. *et al.* Zoonotic transmission of avian influenza virus (H5N1), Egypt, 2006-2009. *Emerg Infect Dis* **16**, 1101–1107 (2010).

31. World Health Organization. Avian influenza – situation in Iraq - Update. https://www.who.int/emergencies/disease-outbreak-news/item/2006_02_02-en.

32. World Health Organization. Weekly Epidemiological Record, 2008, vol. 83, 40. *Weekly Epidemiological Record = Relevé épidémiologique hebdomadaire* https://iris.who.int/handle/10665/241213 (2008).

33. World Health Organization. Avian influenza – situation in Pakistan - update. https://www.who.int/emergencies/disease-outbreak-news/item/2007_12_27-en.

34. Brooks, W. A. *et al.* Avian Influenza Virus A (H5N1), Detected through Routine Surveillance, in Child, Bangladesh - Volume 15, Number 8—August 2009 - Emerging Infectious Diseases journal - CDC. doi:10.3201/eid1508.090283.

35. World Health Organization. Avian influenza weekly update 2025. (2024).

36. World Health Organization. Human infection with avian influenza A(H5N1) ｰ India. https://www.who.int/emergencies/disease-outbreak-news/item/human-infection-with-avian-influenza-a(h5n1)-ｰ-india.

37. Kandun, I. N. *et al.* Three Indonesian clusters of H5N1 virus infection in 2005. *N Engl J Med* **355**, 2186–2194 (2006).

38. Yang, Y., Halloran, M., Sugimoto, J. & Longini, I. Detecting human-to-human transmission of avian influenza a (H5N1). *EMERGING INFECTIOUS DISEASES* **13**, 1348–1353 (2007).

39. Olsen, S. J. *et al.* Family clustering of avian influenza A (H5N1). *Emerg Infect Dis* **11**, 1799–1801 (2005).

40. WHO Western Pacific Region. Avian influenza. https://www.who.int/westernpacific/wpro-emergencies/surveillance/avian-influenza.

41. World Health Organization. Information on Avian Influenza A (H5N1) Identified in Human in Nepal. https://www.who.int/nepal/news/detail/01-05-2019-information-on-avian-influenza-a-(h5n1)-identified-in-human-in-nepal.

42. Ungchusak, K. *et al.* Probable person-to-person transmission of avian influenza A (H5N1). *N Engl J Med* **352**, 333–340 (2005).

43. FluTrackers. Cambodia. *FluTrackers News and Information* https://flutrackers.com/forum/forum/cambodia/cambodia-h5n1-tracking.

44. Lai, S. *et al.* Global epidemiology of avian influenza A H5N1 virus infection in humans, 1997-2015: a systematic review of individual case data. *Lancet Infect Dis* **16**, e108–e118 (2016).

45. Yu, H. *et al.* The first confirmed human case of avian influenza A (H5N1) in the mainland of China. *Lancet* **367**, 84 (2006).

46. Wang, H. *et al.* Probable limited person-to-person transmission of highly pathogenic avian influenza A (H5N1) virus in China. *Lancet* **371**, 1427–1434 (2008).

47. Centre for Health Protection. Avian Influenza Report. https://www.chp.gov.hk/en/resources/29/332.html.

48. FluTrackers Global H5N6 Cumulative Case List. *FluTrackers News and Information* https://flutrackers.com/forum/forum/china-h5n1-h5n8-h5n6-h5n3-h5n2-h10n8-outbreak-tracking/723926-flutrackers-global-h5n6-cumulative-case-list (2015).

49. Centers for Disease Control and Prevention (CDC). Isolation of avian influenza A(H5N1) viruses from humans--Hong Kong, May-December 1997. *MMWR Morb Mortal Wkly Rep* **46**, 1204–1207 (1997).

50. Centers for Disease Control and Prevention (CDC). Update: isolation of avian influenza A(H5N1) viruses from humans--Hong Kong, 1997-1998. *MMWR Morb Mortal Wkly Rep* **46**, 1245–1247 (1998).

51. Peiris, J. S. M. *et al.* Re-emergence of fatal human influenza A subtype H5N1 disease. *Lancet* **363**, 617–619 (2004).

52. Yuen, K. Y. *et al.* Clinical features and rapid viral diagnosis of human disease associated with avian influenza A H5N1 virus. *Lancet* **351**, 467–471 (1998).

53. Puthavathana, P. *et al.* Avian influenza virus (H5N1) in human, Laos. *Emerg Infect Dis* **15**, 127–129 (2009).

54. Sengkeopraseuth, B. *et al.* First human infection of avian influenza A(H5N6) virus reported in Lao People’s Democratic Republic, February-March 2021. *Influenza Other Respir Viruses* **16**, 181–185 (2022).

55. World Health Organization. Avian Influenza A(H5N1) – Viet Nam. https://www.who.int/emergencies/disease-outbreak-news/item/2024-DON511.

56. FluTrackers. Vietnam. *FluTrackers News and Information* https://flutrackers.com/forum/forum/asia/h5n1-h5n8-h1n08-tracking/vietnam-aa.

57. Hien, T. T. *et al.* Avian Influenza A (H5N1) in 10 Patients in Vietnam. *N Engl J Med* **350**, 1179–1188 (2004).

58. de Jong, M. D. *et al.* Fatal avian influenza A (H5N1) in a child presenting with diarrhea followed by coma. *N Engl J Med* **352**, 686–691 (2005).
